# A Comparison between Different Error Modeling of MEMS Applied to GPS/INS Integrated Systems

**DOI:** 10.3390/s130809549

**Published:** 2013-07-24

**Authors:** Alex G. Quinchia, Gianluca Falco, Emanuela Falletti, Fabio Dovis, Carles Ferrer

**Affiliations:** 1 Departament de Microelectrònica i Sistemes Electrònics, Universitat Autònoma de Barcelona (IEEC-UAB), Bellaterra, Barcelona 08193, Spain; E-Mail: carles.ferrer@imb-cnm.csic.es; 2 Institut Politecnico di Torino and Istituto Superiore Mario Boella, via Pier Carlo Boggio 61, Turin 10138, Italy; E-Mails: falco@ismb.it (G.F.); falletti@ismb.it (E.F.); fabio.dovis@polito.it (F.D.); Tel.: +39-011-2276414; 3 Institut de Microelectrònica de Barcelona (CNM, CSIC), UAB, Barcelona 08193, Spain

**Keywords:** Allan variance, power spectral density, INS/GPS, error modeling, MEMS, AR models, wavelet de-noising

## Abstract

Advances in the development of micro-electromechanical systems (MEMS) have made possible the fabrication of cheap and small dimension accelerometers and gyroscopes, which are being used in many applications where the global positioning system (GPS) and the inertial navigation system (INS) integration is carried out, *i.e.*, identifying track defects, terrestrial and pedestrian navigation, unmanned aerial vehicles (UAVs), stabilization of many platforms, *etc.* Although these MEMS sensors are low-cost, they present different errors, which degrade the accuracy of the navigation systems in a short period of time. Therefore, a suitable modeling of these errors is necessary in order to minimize them and, consequently, improve the system performance. In this work, the most used techniques currently to analyze the stochastic errors that affect these sensors are shown and compared: we examine in detail the autocorrelation, the Allan variance (AV) and the power spectral density (PSD) techniques. Subsequently, an analysis and modeling of the inertial sensors, which combines autoregressive (AR) filters and wavelet de-noising, is also achieved. Since a low-cost INS (MEMS grade) presents error sources with short-term (high-frequency) and long-term (low-frequency) components, we introduce a method that compensates for these error terms by doing a complete analysis of Allan variance, wavelet de-nosing and the selection of the level of decomposition for a suitable combination between these techniques. Eventually, in order to assess the stochastic models obtained with these techniques, the Extended Kalman Filter (EKF) of a loosely-coupled GPS/INS integration strategy is augmented with different states. Results show a comparison between the proposed method and the traditional sensor error models under GPS signal blockages using real data collected in urban roadways.

## 1. Introduction

Currently, many land vehicles are equipped with global positioning systems (GPS), which can give an acceptable positioning. However, GPS is affected by several errors (*i.e.*, multipath, ionosphere and troposphere delays), signal unavailability (*i.e.*, momentary blockage while driving through tunnels, indoor car parks or along urban canyons), voluntary or involuntary signal interference, like jamming and spoofing, *etc.* All these errors affect the integrity and reliability of the navigation solution, and only some of them can be reduced or mitigated (*i.e.*, multipath, interference). Others are intrinsic in GPS functioning (*i.e.*, signal blockage and drop in the signal power) and cannot be removed. On the other hand, the inertial navigation system (INS) provides information about position, velocity and attitude with a higher rate than the GPS. It is inherently immune to the signal jamming and blockage vulnerabilities of GPS, but the accuracy of INS is significantly affected by the error characteristics of the inertial sensors [1]. Since these instruments have a complementary nature, the GPS/INS integration provides a higher performance than their stand-alone operation.

Recently, new low-cost and small sized inertial sensors have been built, increasing the demand of the low-cost INS and expanding its use in several applications, where GPS/INS are blended. Nonetheless, low-cost inertial sensors are characterized by high noise and large uncertainties in their outputs, such as bias, scale factor and non-orthogonalities [2]. In other words, a low-cost INS presents errors in position, velocity and attitude, which grow rapidly, degrading the accuracy of the navigation system in a short period of time. Therefore, a suitable modeling of these inertial sensors is necessary in order to improve the system performance.

This paper is focused on the identification and modeling of the bias-drift stochastic error, applying the most used techniques currently available to analyze these random processes. Additionally, since these noises have both high-frequency noise (short-term) and low-frequency noise (long-term), it is necessary to minimize both of them in order to improve the accuracy of the INS. Wavelet de-noising has been used in similar works, because of its great effectiveness removing high-frequency noises, as is shown in [3–6]. However, it has a limited success in removing the long-term inertial sensors errors [7]. Moreover, Allan variance (AV) is a widely used technique in the modeling of inertial sensors, which can take into account the long-term noises [8–11]. Thereby, we present a mixture of the wavelet de-noising technique and Allan variance with the purpose of evaluating the accuracy enhancement of the inertial sensors when these methods are blended together. It is worth mentioning that most of the works where Allan variance is reported are limited to just modeling the stochastic errors of the inertial sensors, and the accuracy of the parameters estimated by the AV are only sometimes tested in real scenarios. Therefore, even though there are works where AV and wavelet de-noising are performed (e.g., [6]), there is not a complete analysis and evaluation under several dynamic conditions when these two techniques are blended together in a GPS/INS-integrated system for land vehicle navigation. In this sense, the contribution of this work is the comparison among some of the most used methods for modeling the stochastic error and a complete analysis for a suitable combination between AV and wavelet de-nosing, including the selection of the level of decomposition. En used in similar works, because of its great effectiveness removing high-frequency noises, as is shown in [3–6]. However, it has a limited success in removing the long-term inertial sensors errors [7]. Moreover, Allan variance (AV) is a widely used technique in the modeling of inertial sensors, which can take into account the long-term noises [8–11]. Thereby, we present a mixture of the wavelet de-noising technique and Allan variance with the purpose of evaluating the accuracy enhancement of the inertial sensors when these methods are blended together. It is worth mentioning that most of the works where Allan variance is reported are limited to just modeling the stochastic errors of the inertial sensors, and the accuracy of the parameters estimated by the AV are only sometimes tested in real scenarios. Therefore, even though there are works where AV and wavelet de-noising are performed (e.g., [6]), there is not a complete analysis and evaluation under several dynamic conditions when these two techniques are blended together in a GPS/INS-integrated system for land vehicle navigation. In this sense, the contribution of this work is the comparison among some of the most used methods for modeling the stochastic error and a complete analysis for a suitable combination between AV and wavelet de-nosing, including the selection of the level of decomposition.

This paper is organized as follows. Firstly, we begin with an introduction to the noises that are involved in a low-cost INS (micro-electromechanical systems (MEMS) grade) (Section 2). Secondly, the architecture employed to integrate INS and GPS data is described, as well as the state-space form of different error models (Section 3). Thirdly, the analysis of the underlying random processes that affect the inertial sensors is achieved by different techniques: autocorrelation, Allan variance (AV), power spectral density (PSD) and autoregressive processes (Section 4). Subsequently, the parameters of various stochastic models are obtained from the methods presented in the previous section by using experimental data collected in the laboratory (Section 5). This section also explains the combination between wavelet de-noising and autoregressive (AR) models with different orders, and the combination between AV and wavelet de-noising techniques using different levels of decomposition. Finally, the models that are identified using AV and PSD, wavelet de-noising/AR models and the proposed method based on wavelet de-nosing/AV are adapted to the loosely-coupled integration, assessed using real data collected in urban roadways and compared (Sections 6 and 7).

## 2. MEMS-Based INS

**Figure 1.**
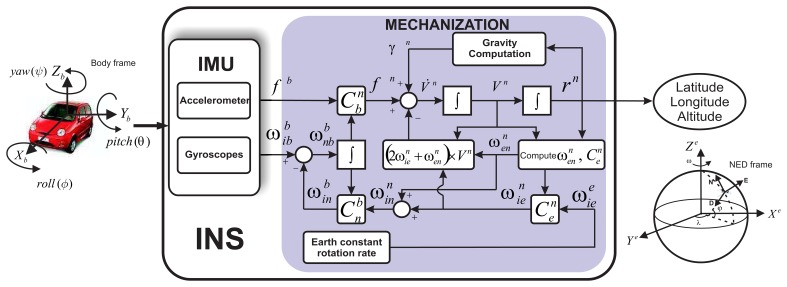
Navigation frame inertial navigation system (INS) mechanization; figure kindly taken from [14].

The strapdown inertial navigation system (INS) involves mechanization equations, which are the numerical tool to implement the physical phenomenon that relates the inertial sensor measurements to the navigation state (*i.e.*, position, velocity and attitude) [10]. The shaded rectangle in Figure 1 represents the INS mechanization equations that can describe the motion of a vehicle, taking as input the inertial measures in the body frame (accelerations and angular rotations) and converting these measurements into a reference frame for navigation. In this case, it provides position, velocity and attitude of the vehicle with respect to the North-East-Down (NED) local geodetic frame. The complete derivation of the navigation equations is reported in [10,12,13].

**Figure 2.**
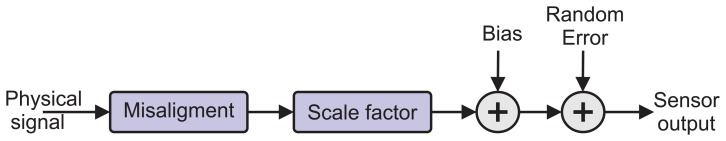
Inertial sensor error modeling; figure kindly taken from [15].

The inertial measurement unit (IMU), which is part of the INS, is the device where the inertial sensors are mounted; it provides the accelerations and angular rotations along three orthogonal directions with respect to the body frame (Figure 1). In a low-cost INS (MEMS grade), the measurement of these accelerometer and gyro sensors is affected by different errors, which can be classified as deterministic and stochastic errors. Figure 2 depicts some of these errors through a simple relationship between IMU physical signal and the sensor output.

Deterministic errors are due to manufacturing and mounting defects and can be calibrated out from the data; on the other hand, the stochastic errors are the random errors that occur due to random variations of bias or scale factor over time [10]. There are several errors that affect the inertial sensors: the misalignment errors are the result of non-orthogonalities of the sensor axes and are usually treated as deterministic error. The scale factor represents the sensibility of the sensor, and it is the result of manufacturing tolerances or aging; it is usually divided between a linear and a non-linear part, where the linear part is obtained from calibration, while the non-linear is modeled with a stochastic process [16]. In the case of the bias, it is divided between bias turn-on and bias-drift: the bias turn-on is constant, but it varies from turn-on to turn-on and is considered as a deterministic error; the bias-drift presents a random behavior and needs to be modeled with a stochastic process [17]. Regarding the random error (Figure 2), this is an additional signal resulting from noise of the sensor itself or other components that interfere with the signal provided by the sensor; it is also considered as part of the stochastic error of the sensor.

An additional factor that also affects the inertial sensors based on MEMS technology is the temperature. However, it will not be covered in this work. For further details of temperature dependence of the stochastic error and the different errors that affect the MEMS sensors, refer to [10,16,18,19].

The deterministic errors can be minimized before implementing the mechanization equations by following different procedures through laboratory calibrations (see [19]). In this work, we focused on the stochastic error, specifically, in the bias-drift, since the stochastic modeling of this error is a challenging task, not only because of the random nature, but also because it seriously affects the performance of a navigation system. For further details of the impact of this error, refer to [20,21], where how the position error grows when different bias-drift are affecting the inertial sensor measurements has been analyzed. Therefore, a suitable estimation of the stochastic model parameters of this error will improve the performance of the INS; as a consequence, the input error to the mechanization stage (Figure 1) can be compensated and, in turn, the position error minimized.

The next section will present some of the stochastic processes that are usually used to model the bias-drift that affects the INS and their state-space representation. We will also explain the loosely-coupled architecture that is addressed to integrate INS and GPS data.

## 3. Loosely-Coupled KF Integration and State Vector Augmentation

### 3.1. Loosely-Coupled KF Integration

It is common to blend GPS and INS using different integration approaches (*i.e.*, loosely-coupled, tightly-coupled or ultra-tightly coupled; see [22–24]). In this paper, we confine our attention in the loosely-coupled (LC) approach, because this strategy can be used to evaluate the behavior of the inertial sensor stochastic model without any additional support during partial or complete GPS outages, which is not the case of the tightly-coupled integration, where one satellite signal available might be used to compute the Extended Kalman Filter (KF; *i.e.*, tightly-coupled uses GPS estimates of pseudoranges and Doppler determined by using satellite ephemeris data). There are two ways to implement the LC strategy: feed-forward and feed-back. The first one is used in systems that have a high-performance inertial measurement unit (IMU), as it merges the GPS/INS information, but it has no control over the error that may occur in the IMU; it basically works with a open-loop architecture.

**Figure 3.**
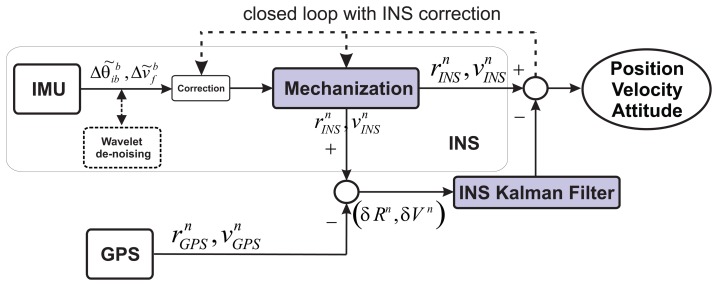
Loosely-coupled Kalman Filter (KF) integration with feedback; figure kindly taken from [25]

On the other hand, the feed-back includes a close-loop that allows us to correct the INS error, where in the case of a GPS outage, the navigation solution will depend only on the INS, which will be corrected by its correspondent inertial sensor error model. The block diagram of the GPS/INS integration with feedback is shown in Figure 3.

In this strategy, the position and velocity obtained from the mechanization $\left(r_{\text{INS}}^{n},v_{\text{INS}}^{n}\right)$ are combined with the GPS, which delivers velocity and position data $\left(r_{\text{GPS}}^{n},v_{\text{GPS}}^{n}\right)$. The residual error (*δR^n^*,*δV^n^*) calculated from the GPS and INS outputs is the input to the Kalman Filter (KF), where a state-space model is built with error states for navigation and IMU errors. The error states related to the IMU errors are fed back though the closed loop in order to correct the INS navigation solution.

The system model for loosely-coupled approach is given by position error, velocity error and attitude error, which represent the navigation error states, *i.e.*, a total of nine states for 3D navigation. Moreover, the scale factors and bias for gyro and accelerometers are included in the IMU error states, and the number of states will depend on the stochastic model employed.

The next section will describe the stochastic processes that will augment the state-space model with the IMU error states associated to the inertial sensor bias-drift. The corresponding stochastic model for each error state will be selected in Section 6, after having analyzed the inertial sensors data with the techniques that will be explained in Section 4. For further details about the navigation error states for the loosely-coupled integration, refer to [10,13,23].

### 3.2. State-Space Representation for Different Bias Models

Various stochastic processes are well detailed in [26-28]. In this section, we will focus on the ones that will be used to augment the LC integration.

The bias-drift models adapted into the LC approach are the first order Gauss-Markov, random walk and autoregressive processes, which are a generalized representation of the first mentioned one.

First order Gauss-Markov (GM): This process has been widely used for modeling random errors, not only because it is able to represent a large number of physical processes, but also because it has a relatively simple mathematical description [29]. The continuous model for this process is described by the following equation:

(1) $$\dot{x}=-\frac{1}{T_{c}}x+w$$

where *x* is a random process with zero mean, correlation time, *T_c_*, and driven noise, *w*. The corresponding discrete time equation can be written as:

(2) $$x_{k}=(1-\beta \Delta t)x_{k-1}+w_{k}$$

where Δ*t* is the sampling time and *w_k_* is a white noise with noise covariance:

(3) $$\sigma_{w_{k}}^{2}=\sigma_{x_{k}}^{2}(1-e^{-2\Delta t/T_{c}})$$

where $\sigma_{x_{k}}^{2}=\sigma_{GM}^{2}$ is the covariance of the process. The continuous time representation of the noise covariance can be expressed as:

(4) $$\sigma_{w}^{2}=2\beta \sigma_{GM}^{2}$$

where *β* is the inverse of the correlation time, *T_c_*. Once the correlation time (*T_c_*) and the covariance of the process $\left(\sigma_{x_{k}}^{2}\right)$ are obtained by Allan variance (see Section 4.4), the model of the first order GM process can be implemented as a state-space in Extended Kalman Filter (EKF), either with Equation (1) or Equation (2), depending on if the transition matrix is either in continuous or discrete time.

Random walk (RW): This process results when uncorrelated signals are integrated, e.g., when white noise is integrated during the mechanization stage. The continuous and discrete time of the RW are represented by:

(5) $$\dot{x}=w$$

(6) $$x_{k}=x_{k-1}+w_{k}$$

where *w* is a white noise with noise covariance *q_k_* = *q*(*t_k_*_+1_ – *t_k_*) = *σ_RW_*^2^Δ*t*. The uncertainty of the random walk increases with time; therefore, it is a non-stationary process [26]. However, it can be considered stationary within small time intervals [30].

The noise covariance of the RW process can be obtained from power spectral density or Allan variance analysis, which will be described in Sections 4.3 and 4.4, respectively. This process is used to represent rate/acceleration random walk (K).

A typical bias-drift of a inertial sensor can be represented by a combination of different random processes, such as white noise (WN), RW and first order GM processes. These processes can be added into the KF by writing them in a state-space model. According to the previous definitions, a random process that combines WN, RW and first order GM can be generated using the following discrete time-invariant state-space model:

(7) $${\left(\begin{array}{c} x_{1}\\ x_{2} \end{array}\right)}_{k}=\left(\begin{array}{cc} \left(1-\beta \Delta t\right) & 0\\ 0 & 1 \end{array}\right)\;{\left(\begin{array}{c} x_{1}\\ x_{2} \end{array}\right)}_{k-1}+\left(\begin{array}{c} \sigma_{GM}\sqrt{\left(1-e^{-2\Delta t/T_{c}}\right)}\\ \sigma_{RW}\sqrt{\Delta t} \end{array}\right)w_{k}$$

(8) $$y_{k}=\left(\begin{array}{cc} 1 & 1 \end{array}\right){\left(\begin{array}{c} x_{1}\\ x_{2} \end{array}\right)}_{k}+\left(\sigma_{WN}/\sqrt{\Delta t}\right)v_{k}$$

where *σ_WN_* is the standard deviation of the white noise process and *y_k_* is the result of combining WN, RW and first order GM. Equations (7) and (8) are easily adapted into the KF equations, since they are represented in state-space form. In this example, the bias-drift (*y*) would be modeled by the combination of three noises, *i.e.*, *y* = *WN* + 1*^st^GM* + *RW*.

Autoregressive (AR) process: An AR process is a time series produced by linear combination of past values, which can be described by the following linear equation [31]:

(9) $$x(n)=-\sum_{k=1}^{p}\alpha_{k}x(n-k)+\beta_{0}w(n)$$

where *x*(*n*) is the process output, which is a combination of past outputs, plus a white noise, *w*(*n*), with standard deviation, *β*_0_; *p* is the order of the AR process and *α_k_* are the model parameters.

In order to include the AR process in the EKF transition matrix, it is necessary to express Equation (9) in state-space form. If we consider a third order AR process, the corresponding state-space form can be expressed as follow [29]:

(10) $${\left(\begin{array}{c} x_{1}\\ x_{2}\\ x_{3} \end{array}\right)}_{n}=\left(\begin{array}{ccc} 0 & 1 & 0\\ 0 & 0 & 1\\ -\alpha_{3} & -\alpha_{2} & -\alpha_{1} \end{array}\right)\;{\left(\begin{array}{c} x_{1}\\ x_{2}\\ x_{3} \end{array}\right)}_{n-1}+\left(\begin{array}{c} 0\\ 0\\ \beta_{0} \end{array}\right)\;w(n)$$

This represents the AR model in state-space for one of the inertial sensors. It should be noted that if the order of the AR model increases by one, the variables in the state vector of the Kalman filter will increase by six, since this model is applied to each axis of inertial sensors.

The stochastic processes that are used to model the inertial sensors bias-drift are augmented into the Kalman filter, as was explained in this section. In order to obtain the parameters of each stochastic process, an analysis of the sensors data needs to be done. The methods addressed to get these parameters are discussed in Section 4, and the experimental analysis of each method is presented in Section 5.

## 4. Identifying and Extracting Stochastic Model Parameters

The stochastic modeling of the inertial sensors is a challenging task that in most practical cases, is performed by tuning the GPS/INS Extended Kalman Filter, which is often sensitive and difficult, by using sensors available specifications, but low-cost sensors do not provide enough information to develop this sort of models, or by experience [32]. Therefore, there are different works that have been achieved in order to obtain a suitable estimation of the stochastic model parameters [5,6,8,9,33,34]. In this section, we describe the most used methods for noise identification and extraction of the noise parameters for stochastic modeling of inertial sensors. Additionally, an introduction to the wavelet de-noising technique is presented at the end of the section.

### 4.1. Autocorrelation

The autocorrelation function has been used in previous works to analyze the stochastic error of the inertial sensors [5,33] and also to obtain the parameters for modeling, using the first order Gauss-Markov (GM) process. As it was explained in the previous section, this process seems to fit a large number of physical processes with reasonable accuracy.

**Figure 4.**
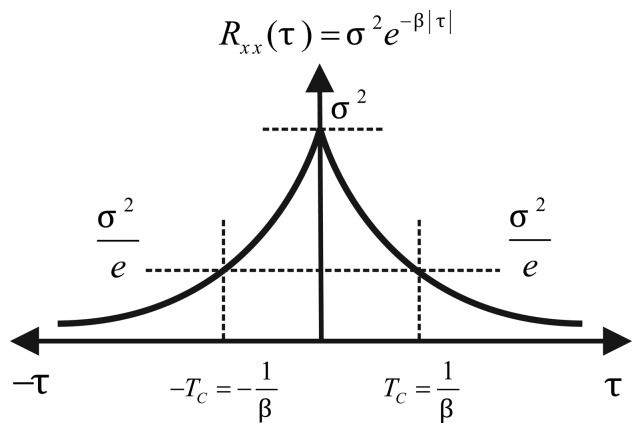
The autocorrelation function of the first order Gauss-Markov process.

For a random process, *x*, with zero mean, correlation time, *T_c_*, and driven noise, *w*, the first order Gauss-Markov (GM) process is described by Equation (1). The parameters needed to implement this process can be extracted from its autocorrelation function (Figure 4), which is given by:

(11) $$R_{\text{xx}}(\tau )=\sigma^{2}e^{\beta \left|\tau \right|}$$

where the correlation time is *T_c_* = 1/*β* and *σ*^2^ is the variance of the process at zero time lag (*τ* = 0). The most important characteristic of the first order GM process is that it can represent bounded uncertainty, which means that any correlation coefficient at any time lag, *τ*, is less or equal the correlation coefficient at zero time lag, *R_xx_* (*τ*) ≤ *R_xx_*(0) [26].

One of the limitations of this method is that an accurate autocorrelation curve from experimental data is rarely done, due to the fact that the data collected is limited and finite. As it is discussed in [29], the accuracy of the autocorrelation depends on the recorded length data.

In [5,33,34], it was shown that the autocorrelation function of experimental inertial sensor data might not be as a first order GM process, which is equivalent to a first order autoregressive process. This means that only a first order autoregressive process may not be adequate to model the bias-drift behavior that affects the performance of the inertial navigation system. In fact, in most of the cases when low-cost IMU are used, the shape of the autocorrelation follows higher order Gauss-Markov processes. As a consequence, higher orders of autoregressive processes are more appropriate to model inertial sensors stochastic errors [35]. Despite this, the autocorrelation analysis can be useful to determine the correlation grade of the underlying random processes that affect the sensors and, also, if the uncorrelated noise can be removed after filtering the sensor signal. This issue will be discussed in Section 5.2.

### 4.2. Autoregressive Processes

To avoid the problem of inaccurate modeling of inertial sensor random errors, as in the case with the low-precise autocorrelation function, described in Section 4.1, another method, which was introduced in [5], can be applied. There are different works where the autoregressive (AR) models have been evaluated, some of them are well detailed in [5,31,33,34].

Although first order Gauss-Markov (GM) process has been very useful for modeling random errors of inertial sensors, better stochastic modeling can be achieved by modeling these errors as higher order AR models [33]. In addition, the autocorrelation of the random error for MEMS sensors often seems to follow a higher order GM process, which can be modeled using an appropriate AR model.

According to Equation (9), this is assumed that the coefficients (*β*_0_, *α_k_*) are computed so that the linear system is stable, making the model stationary [26]. It should be noted in Equation (9) that if *p* = 1, then the AR process approximates first order GM processes. On the other hand, if *p* = 1 and *α*_1_ = −1, it becomes a random walk (RW), and if *α*_1_ = 0, it would be a white noise (WN). The coefficients of this process are estimated by Burg's method, since it overcomes some of the drawbacks of other methods by providing more stable models and improved estimates with shorter data records [36].

In this paper, we focus on AR models up to the third order, since a higher order would increase the computational load and might result in unstable solutions [5]. This method is usually used after applying wavelet de-noising to the static inertial sensor data, which is explained in Section 4.5.

### 4.3. Power Spectral Density

Power spectral density (PSD) is an important descriptor of a random process, because it provides information of the signal that is not easy to extract from the time domain.

The PSD is related to the autocorrelation function with:

(12) $$S_{x}(jw)=F[R_{xx}(\tau )]=\int_{-\infty }^{\infty }R_{xx}(\tau )e^{-\text{jwt}}d\tau$$

where, *S_x_*(*jw*) is the power spectral density of the process, *x*,ℱ [·] indicates Fourier transform, and *R_xx_*(*τ*) is the autocorrelation of the process, *x* [37].

Basically, the PSD is used to identify the stochastic errors of the inertial sensors from the frequency components, and the parameters obtained from the PSD are eventually used in the stochastic model of the INS.

**Figure 5.**
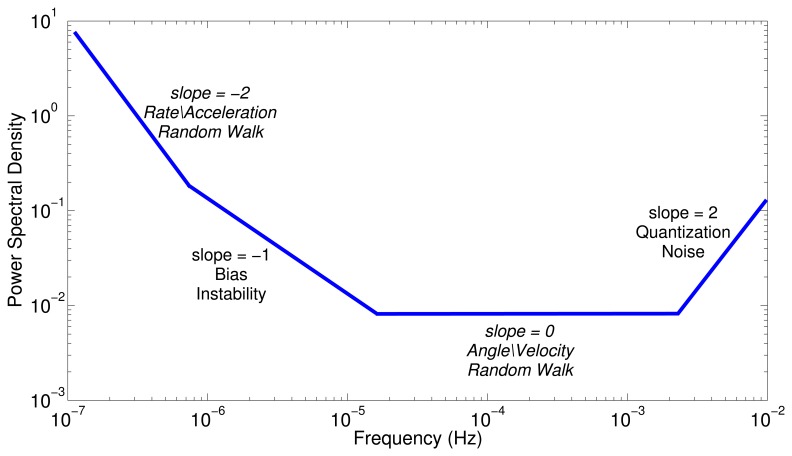
Hypothetical power spectral density (PSD) in single-sided form of an inertial sensor; PSD plot from the IEEE Std 1293-1998 [37].

Figure 5 depicts a hypothetical inertial sensor PSD in single-sided. According to this curve, the noise sources might be identified considering the slopes, *i.e.*, a slope of −2 represents the rate\acceleration random walk noise for gyro and accelerometer, respectively. Obviously, the number of random noises that might be present in the curve depends on the type of sensors. The noise terms that can be identified with the PSD are well detailed in [8,11,37].

Although it is not covered in this paper, it should be mentioned that recently, a more effective analysis in the frequency-domain has been presented by El-Diasty and Pagiatakis, where a GPS/INS impulse response model that is applied in the bridging GPS outages using as input the INS-only navigation solution is developed; for further details about this method, refer to [38].

So far, we have presented the autocorrelation, where the stochastic model parameters are extracted from the autocorrelation curve, the autoregressive processes that estimates the coefficients of an AR model applying Burg's method over the de-noised sensor data and the power spectral density that identifies the noise terms based on the slopes in a log-log PSD curve. The following section will describe the Allan variance technique, which is similar to the PSD, but in the time domain.

### 4.4. Allan Variance

The Allan variance (AV) is a time domain analysis technique originally developed to study the frequency stability of oscillators [39]. More recently, this has been successfully applied to the modeling of inertial sensors [6,19,39–41], and two key documents to determine the characteristics of the random processes that give rise to the measurement noise of the sensors using this technique are [8,11]. As such, AV helps in identifying the source of a given noise term in the observed data [11].

The Allan variance is estimated as follows:

(13) $$\sigma^{2}(T)=\frac{1}{2T^{2}(N-2n)}\sum_{k=1}^{N-2n}{\left(\theta_{k+2n}-2\theta_{k+n}+\theta_{k}\right)}^{2}$$

where *T* represents the correlation time, or cluster time, *i.e.*, the time associated with a group of *n* consecutive observed data samples, *N* is the length of the data that will be analyzed and *θ* is the output velocity, in the case of the accelerometers, and output angle, in the case of the gyros; these measurements are made at discrete times from the inertial sensors.

The basic idea to estimate the AV is to take a long sequence of data (*N*), where the IMU is in a static condition. After having removed the turn-on bias from the gyros' and accelerometer's stored data, the output of the inertial sensor is integrated to get *θ*. Thus, the AV can be computed through Equation (13).

**Figure 6.**
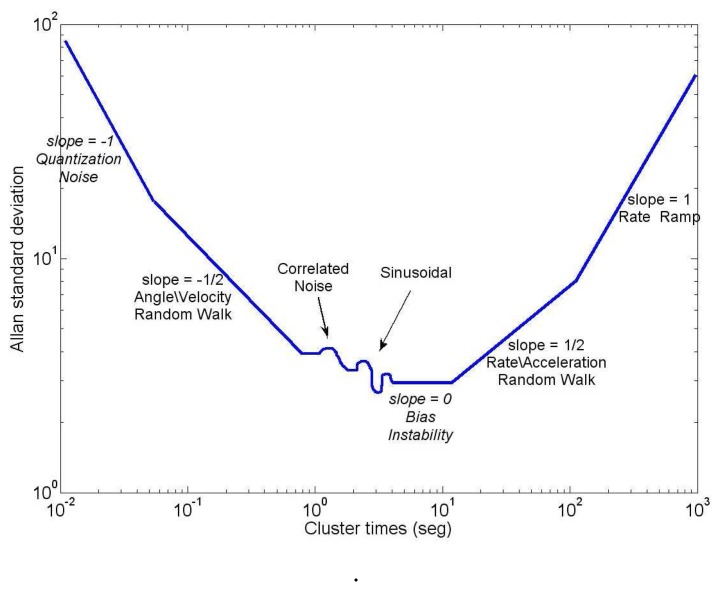
Hypothetical Allan variance (AV) of an inertial sensor; AV plot from the IEEE Std 952-1997 [11]

In AV, the uncertainty in the data is assumed to be generated by noise sources of specific character, as for instance, rate random walk, angle random walk, bias instability, *etc.* In order to obtain the covariance of each noise source affecting the sensor output, it is necessary to analyze the computed AV result by Equation (13). This is usually achieved by plotting a log-log AV curve, as is depicted in Figure 6, from which the covariance values for each error can be extracted doing a similar analysis to the one performed with the PSD curve.

The AV obtained from Equation (13) is related to the two-sided PSD by:

(14) $$\sigma^{2}(T)=4\int_{0}^{\infty }df\cdot S_{x}(f)\cdot \frac{\sin^{4}(\pi fT)}{{\left(\pi fT\right)}^{2}}$$

where *S_x_*(*f*) is the PSD of the random process, *x*, written in Equation (12).

An interpretation of Equation (14) is that the Allan variance is proportional to the total noise power of the sensor output when passed through a bandpass filter with transfer function sin^4^(*πfT*)/(*πfT*)^2^. This filter depends on *T*, which suggests that different types of random processes can be examined by adjusting the correlation time (*T*). Thus, the AV provides a mean of identifying and quantifying various noise terms that exist in the data [11].

Computation of AV needs a finite number of clusters that can be generated from the raw data measurements of the sensors. Depending on the size of these clusters, AV can identify any noise term that is affecting the data sensor. It is important to mention that the estimation accuracy of the AV for a given *T* depends on the number of independent clusters within the data set [11]. The bigger the number of independent clusters, the better the estimation accuracy. It has been described in [8] that the percentage error of AV, σ(*δ*), in certain *σ*(*T*) and with a data set of N points is given by:

(15) $$\sigma (\delta )=\frac{1}{\sqrt{2\left(\frac{N}{n}-1\right)}}$$

where *N* is a set of data points collected from the sensors and *n* is the number of data points of the cluster in estimating *σ*(*T*). Equation (15) shows that the estimation errors in the region of short cluster length, *T*, are small, as the number of independent cluster in these regions is large. On the other hand, the estimation error in the region of long cluster length, *T*, are large, as the number of independent clusters in these regions is small [8,11].

For example, if 360,000 data points are collected from an inertial sensor and if we want to compute the estimation accuracy of the AV for a bias instability (Figure 6) with a characteristic time of 10 min, we will have 60,000 points with a sampling frequency of the sensor equal to 100 Hz. According to Equation (15), the percentage error of the AV for this random process would be approximately 32%.

The following section presents wavelet de-noising technique, which will be combined with autoregressive processes, as well as Allan variance.

### 4.5. Wavelet De-Noising

**Figure 7.**
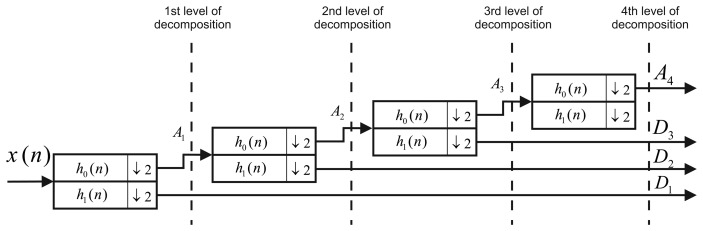
Filter banks of the discrete wavelet transform.

**Figure 8.**
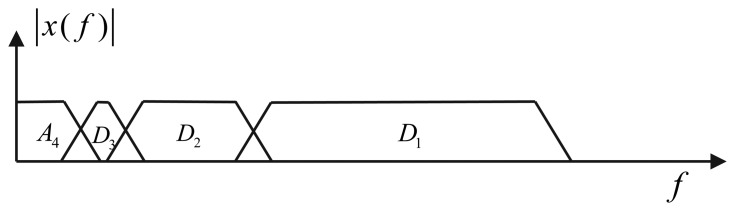
Band frequency distribution after applying four levels of decomposition.

The Discrete Wavelet Transform (DWT) is a widely used technique in digital signal processing, and one of its characteristics is that allows us to do a multiresolution analysis. Basically, when DWT is applied to a signal, *x*(*n*), this is filtered with low-pass, *h*_0_(*n*), and high-pass, *h*_1_(*n*), filters (the coefficients of each filter depend on the wavelet function). Subsequently, a sub-sampling by two is done. Wavelet multiple levels of decomposition (LOD) are obtained by repeating this stage on the sub-sampled output of the low-pass filter, *h*_0_(*n*), as shows Figure 7. After applying DWT, the spectrum of the signal, *x*(*n*), is divided into different sub-bands with different resolutions, as can be seen in Figure 8. The most significant coefficients of the signal, *x*(*n*), are the approximations (*A_k_*). This means, that they have the majority of the information of the signal, while the high-frequency components are know as details (*D_k_*), and as its name says, they are details of the signal, *x*(*n*), that in most cases, are high-frequency noise components.

Moreover, wavelet de-noising takes advantage of the sub-band decomposition performed by the DWT and removes the noise by eliminating the frequency components that are less relevant; in general, this procedure is called wavelet de-nosing and is well described in [3,35,42,43].

This technique is the current state-of-the-art technique used in the accuracy enhancement of inertial sensors [3–5,7]. Since inertial sensors are composed by long-term and short-term noises, wavelet de-noising can be applied in order to remove part of the high-frequency components (short-terms noises). Although wavelet de-noising of INS sensors has had limited success in removing both noise components, it has been combined with AR processes and the autocorrelation function by using the inertial sensor measurements in static conditions. Basically, when it is applied in the autocorrelation method, the uncorrelated noise is removed using wavelet de-noising in order to obtain a smooth autocorrelation function that can be associated to a stochastic process. In the case of the AR process, wavelet de-noising is applied, and then the AR coefficients are estimated from the residual noise.

Wavelet de-noising might be used to remove long-term noises (low-frequency) by increasing the level of decomposition that at the same time, increases the number of frequency bands that can be de-noised. However, in land-vehicle applications, these low-frequency components consist not only of long-term noises, but also vehicle motion dynamics. Since wavelet de-noising can be used to remove the high-frequency components and the AV method can be used to model the long-term noises without removing the vehicles motion, these two methods are combined in order to enhance the INS accuracy. The mixture between these two techniques is addressed in the following section, as well as the experimental analysis for each method explained.

## 5. Experimental Analysis

### 5.1. Inertial Measurement Unit and Data Acquisition

**Figure 9.**
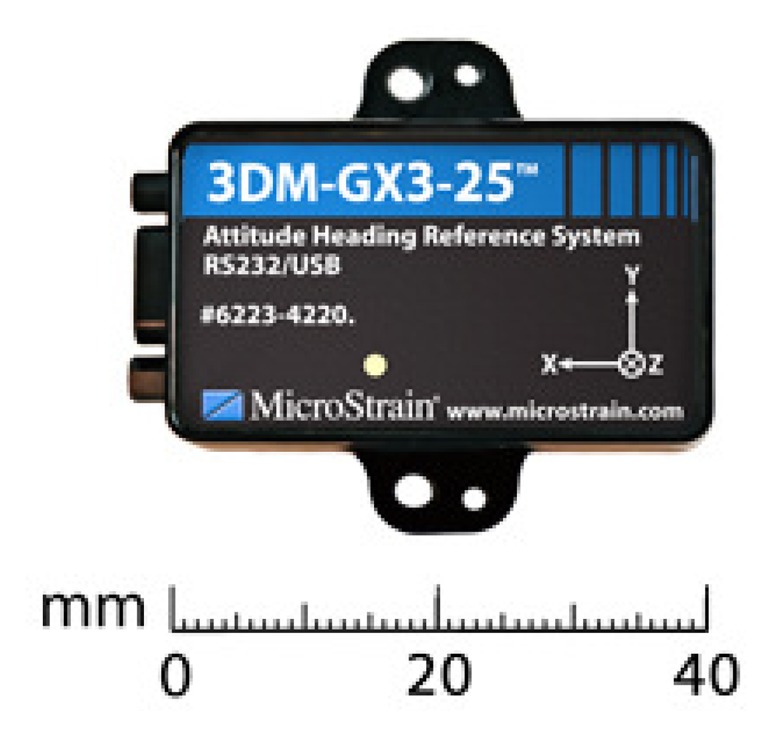
3DM-GX3-25 inertial measurement unit (IMU).

In order to evaluate and compare the previous methods, the static data for analysis was obtained from the IMU 3DM-GX3-25 MEMS grade of MicroStrain (Figure 9). It combines a triaxial accelerometer, triaxial gyro, triaxial magnetometer and temperature sensors. It also includes analog anti-aliasing filters, which accomplish a noise filtering. This stage is followed by a two stage digital moving average filter [44], and the on-board processor of the IMU 3DM-GX3-25 implements these filter stages. Additionally, all quantities are temperature compensated and are mathematically aligned to an orthogonal coordinate system.

The IMU was configured with a sampling frequency of 100 Hz, and the second moving average filter stage implemented in the microcontroller was adjusted with a filter width of 15; this means an attenuation of 14.16% at 20 Hz; for further details of this digital filter, which is embedded on the IMU, see [45].

The characteristics provided by the manufacturer can be seen in Table 1. The test for static analysis was conducted in a room temperature at the Navsas laboratory, Politecnico di Torino [46]. Seven hours of static data were collected in order to analyze the inertial sensors data with the methods that were explained previously. The following sections provide details of the analysis achieved for this IMU data.

### 5.2. Autocorrelation Analysis

**Figure 10.**
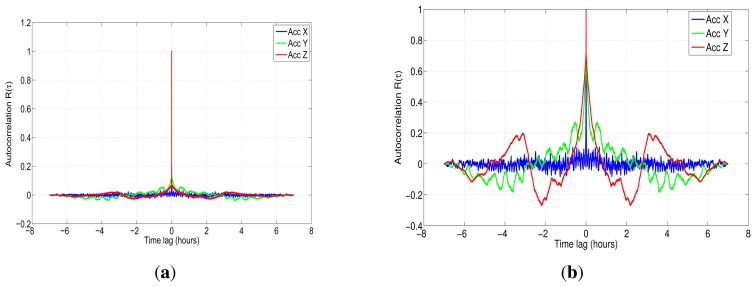
**(a)** IMU 3DM-GX3-25 autocorrelation for accelerometers; **(b)** IMU 3DM-GX3-25 autocorrelation for accelerometers after applying wavelet de-noising with six levels of decomposition (LOD).

After the seven hour-length data collecting, we used the autocorrelation method to achieve the analysis of the random errors that affect the accelerometers and gyroscopes of the IMU. Nevertheless, before processing the raw samples, we removed the turn-on bias for each sensor. Then, the high-frequency terms were attenuated by applying the wavelet de-noising technique. The idea in this step is to minimize the uncorrelated noise that is present in the sensors. Subsequently, the autocorrelation is calculated (Figure 10(b)), and the corresponding parameters should be extracted from the curve. In the case of the first order GM process, they would be stated as *T_c_* and *σ*, respectively.

Figure 10(a) depicts the normalized autocorrelation function of the accelerometers before applying de-nosing, while Figure 10(b) corresponds to the autocorrelation curve after de-noising with six levels of decomposition using Daubechies 4 as the wavelet function. This autocorrelation shows clearly that the residual noise of the *x*-axis accelerometer after applying wavelet de-noising is still dominated by terms that are uncorrelated. With respect to the other two-axes accelerometers (*i.e.*, *y*-axis and *z*-axis), their correlations seems to have more correlated terms than in the *x*-axis accelerometer case, so a high order autoregressive model could be used to model their residual noise, since the autocorrelation curve is similar to the curve of high order AR processes (see [5,26]).

**Figure 11.**
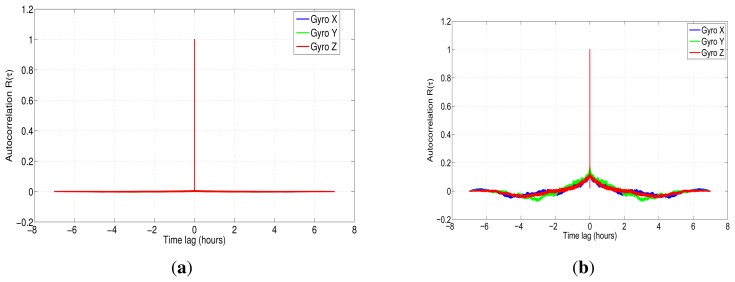
**(a)** IMU 3DM-GX3-25 autocorrelation for gyros; **(b)** IMU 3DM-GX3-25 autocorrelation for gyros after applying wavelet de-noising with six LOD.

The same wavelet de-noising procedure was repeated to analyze the gyroscope's characteristics. The results are depicted in Figure 11(a). This curve shows that the signal for the three gyroscopes is mainly dominated by short-term noises (high-frequency components), which are related to white noise. After applying wavelet de-noising with six levels of decomposition using Daubechies 4 as the wavelet function (Figure 11(b)); the autocorrelation shows that the three gyros have similar characteristics, and although part of the uncorrelated noise was removed, the remaining signal for the gyroscopes still has a representative white noise component.

In the case of inertial sensors based on MEMS technology, the assumption that the stochastic error follows a first order Gauss-Markov process is not valid in most of the situations. This can be visible by comparing Figure 4 with Figure 10(b) and Figure 11(b), where it can be seen that they are different from the autocorrelation function of the first order Gauss-Markov process. This is because these sensors are composed by more complex noise types, and first order Gauss-Markov is only a rough approximation of this complex structure of noises. Nonetheless, for the sake of comparison with the different models and to validate this analysis, a first order AR process is also assessed in Section 7.

It is worth mentioning that the uncorrelated noise could be minimized by applying more levels of decomposition during the wavelet de-noising procedure, or a very high order autoregressive model could be used to create the model. However, the use of such a complex AR model in the integration filter would drastically increase the matrices sizes, as well as the computational burden. In addition, due to the fact that the autocorrelation has some other limitations (see Section 4.1), the method that will be analyzed in the following section is more appropriate to model higher order autoregressive processes.

### 5.3. AR Models

Since the autocorrelation is a low-accurate technique to identify the noises affecting a low-cost INS, a method based on AR models have been used to overcome this issue (see [5]). It consists in combining AR processes and wavelet de-noising to reduce high-frequency noise and, consequently, to obtain the AR coefficients from the residual noise. In other words, after minimizing the short-term error (high-frequency components) with wavelet de-noising, the residual noise could be modeled by an AR model.

For static drift data of the inertial sensors, the approximation part of the DWT includes the earth gravity, the earth rotation rate frequency components and the long-term error, while the detail part of the DWT contains the high-frequency noise and other disturbances [5,43].

By working with inertial data collected in a stationary condition, we first applied the wavelet de-noising technique, and then, the AR model coefficients were estimated with Burg's method. This procedure is executed for each sensor and for two AR models: first and third order. In this work, the attention is focused on these two models, because the first order AR models is one of the most used in the navigation field, and also up to the third order, because as it is explained by Nassar *et al.* [5,34], the higher order would increase the computational load and might result in unstable solutions.

Table 2 depicts the parameters obtained with Burg's method for each inertial sensor using the wavelet de-noising characteristics described in the previous section. It shows the coefficients for the first and third order AR process that correspond to the stochastic process explained in Section 4.2. These AR model coefficients are estimated after computing wavelet de-noising in stationary conditions, which was described in Section 4.5.

### 5.4. PSD Analysis

**Figure 12.**
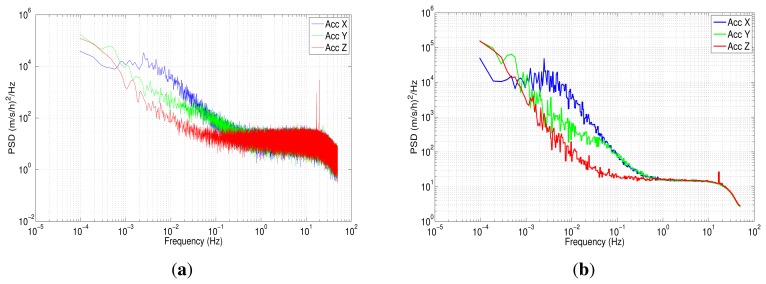
**(a)** Power spectral density accelerometer IMU 3DM-GX3-25; **(b)** power spectral density accelerometer IMU 3DM-GX3-25 after frequency averaging.

The power spectral density was implemented using Welch's method, since this has been found to have the widest application in engineering and experimental physics [47]. In this case, we have applied a Fast Fourier Transform with 2^20^ data points for the seven hours of the data collection. The results for the PSD are shown in Figure 12(a) for accelerometer data.

Figure 12(a) depicts the one-sided PSD for accelerometers data. This log-log plot shows a bunch of high-frequency components, which makes it difficult to identify noise terms and obtain parameters of the stochastic model. The variance in these short-terms noises may be decreased by averaging adjacent frequencies of the estimated PSD [17]; this task can be accomplished by using a technique that is called frequency averaging; further details of this technique can be found in [37]. Figure 12(b) shows a PSD curve after applying frequency averaging; it can be noticed that the noise term identification is easier than in Figure 12(a), and although the low-frequency part of the PSD plot has a high uncertainty, it still conveys some information [37]. According to Figure 5, which was presented in Section 4.3, there are three types of noise: the acceleration random walk (K), the bias instability (B) and the velocity random walk (N). Figure 12(b) shows that the *z*-axis accelerometer has a bias instability (slope −1) smaller than the other two accelerometers, and the velocity random walk is almost the same for all the accelerometers (slope 0).

**Figure 13.**
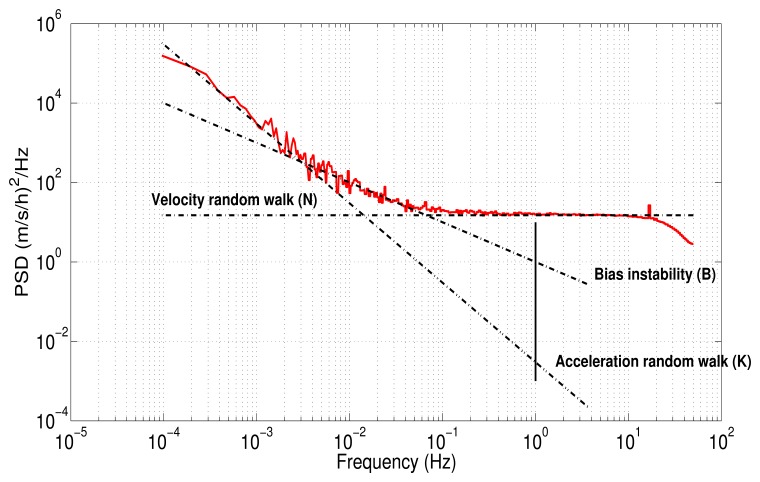
Power spectral density accelerometer Z IMU 3DM-GX-25.

The values for each noise parameter (B,N,K) were extracted drawing straight lines for each frequency band influenced by the noise. The interception of each line with a specific point was taken into account. For instance, the PSD curve for the *z*-axis accelerometer is plotted in Figure 13; it also includes straight dotted lines for each noise, N, B and K, with their respective slopes, 0, −1, −2. The acceleration random walk (K) is present in the low-frequency components between 1 × 10^−4^ Hz and 2.29 × 10^−3^ Hz. This parameter is obtained by fitting a straight line with a slope of −2, starting from 1 × 10^−4^ Hz, until it meets the vertical line of *f* = 1 Hz. Thus, the acceleration random walk for the *z*-axis accelerometer is determined as:

(16) $$k=14.60\;(m/s/h^{3/2})$$

For details of the intercepts to determine the noise parameters, see [11,37]. The bias instability (B) is the dominant noise between 2.29 × 10^−3^ Hz and 7.1 × 10^−2^ Hz, with a slope of −1, while the velocity random walk (N) is present between 0.1248 Hz and 20 Hz. After 20 Hz, there is an attenuation, because of the digital moving average filter, which is used to minimize high-frequency spectral noise produced by the MEMS sensors.

**Figure 14.**
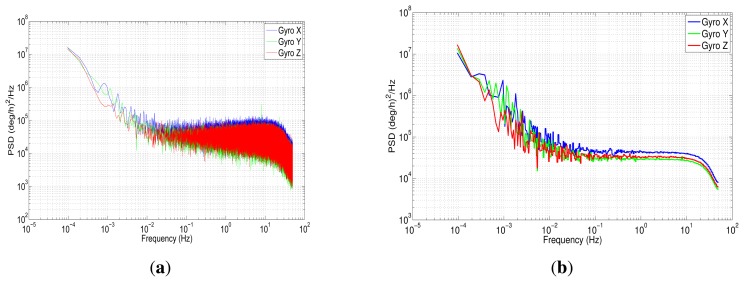
**(a)** Power spectral density gyro IMU 3DM-GX3-25; **(b)** power spectral density gyro IMU 3DM-GX3-25 after applying frequency averaging.

Regarding the gyroscopes, Figure 14(a) represents the power spectral density, while Figure 14(b) corresponds to the gyros PSD after applying frequency averaging; in the latter, was identified as angle random walk (N) and bias instability (B), following the same procedure as with the accelerometers.

Table 3 summarizes the values of different errors that affect the inertial sensors using PSD method. In order to check the validity of these noise coefficients obtained with the power spectral density, AV analysis is presented in the following section.

### 5.5. AV Analysis

**Figure 15.**
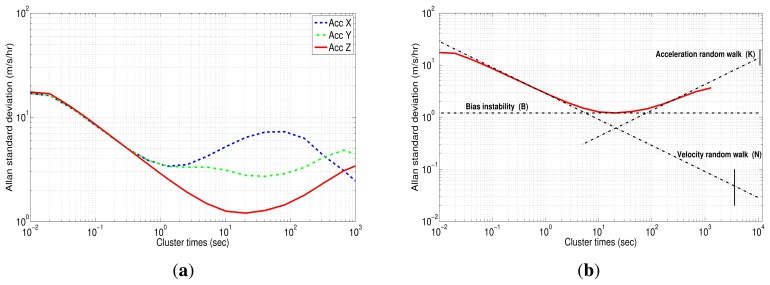
**(a)** IMU 3DM-GX3-25 Allan variance for accelerometers; **(b)** IMU 3DM-GX3-25 Allan variance for accelerometer Z.

**Figure 16.**
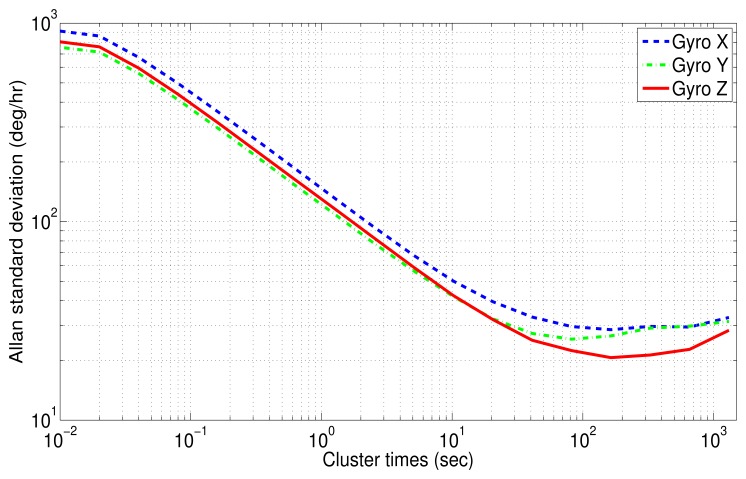
IMU 3DM-GX3-25 Allan variance for three gyro axes.

For Allan variance analysis, the acceleration and the angular rate were integrated to obtain the instantaneous velocity and angle. Subsequently, the log-log plot of Allan variance standard deviation versus cluster times (*T*) was obtained after evaluating Equation (13). The results are plotted in Figure 15(a) for the accelerometer and Figure 16 for gyro data.

Figure 15(a) shows the AV estimated on the 3DM-GX3-25 accelerometers. According to Figure 6, which was presented in Section 4.4, the accelerometers are affected by three types of error: velocity random walk (N), bias instability (B) and acceleration random walk (K). It confirms that *z*-axis accelerometer has a bias instability (slope 0) smaller than the other two accelerometers, and the velocity random walk is almost the same for all the accelerometers (slope −1/2), which is coherent with the results obtained with the PSD.

The values for each noise parameter were extracted as in the PSD, drawing straight lines for each error with its corresponding slope, but in this case, the interceptions are different. To clarify, Figure 15(b) depicts straight lines for each noise of the *z*-axis accelerometer. In this case, the accelerometer has N, B and K with slopes −1/2, 0 and 1/2, respectively. It can be seen that the dominant noise in short cluster times is the velocity random walk, while the dominant error in long cluster times is the acceleration random walk. From the straight line with slope −1/2 fitted to the beginning of the N noise, a value, *σ* = 0.047 (m/s/h), at a cluster times of 1 h can be read. Since the velocity random walk (N) is present in a cluster time interval where the number of independent clusters is very large, the estimation accuracy of the AV is approximately 1.1%. Thus, the velocity random walk or, in other words, the noise term (N) for the z-axis accelerometer is determined as:

(17) $$N=0.047\pm 0.00050\;(m/s/\sqrt{h})$$

The Allan variance standard deviation *versus* cluster times (*T*) for gyro data is depicted in Figure 16. Unlike accelerometers, the gyroscopes have all similar characteristics, where two types of noises can be recognized: angle random walk (N) for short cluster times and bias instability (B) for long cluster times.

For the *x*-axis gyro (blue curve), the bias instability is present in the time range between 321.92 (s) and 654.01 (s). The value of this error can be measured with a flat line at 29.57 (deg/h). Dividing this standard deviation by the factor 0.664, as suggested in [11], the *B* coefficient can be achieved:

(18) B=44.533±5.14(deg/h)

For further details of the intercepts of each noise term in the log-log AV curve, see [8,11,16].

Table 4 summarizes the error coefficients with their respective uncertainty for accelerometers and gyro data. The correlation time, (*T_c_*), of the bias instability, (*B*), and the standard deviation for each sensor (STD) of the IMU 3DM-GX3-25 are shown in Table 5. The correlation time, (*T_c_*), might be used in Equation (1) for modeling the bias instability (B) as a first order Gauss-Markov process; this value is obtained from the segment of the curve where the bias instability is the dominant noise, *i.e.*, the flat segment of the log-log Allan variance curve. It should be mentioned that not only these parameters, but also the whole parameters obtained from AV need to be manually tuned in the KF, since the values obtained from AV are considered an initial approximation of the bias-drift [48].

This verifies the results that were obtained with PSD analysis, where velocity random walk (N), bias instability (B) and acceleration random walk (K) for accelerometers data and angle random walk (N) and bias instability (B) for gyro data were also identified. It can be seen that most of the estimated values in PSD (see Table 3) are within the confidence interval computed by AV (Table 4).

The next section presents the inertial sensor error model that mixtures of AV and wavelet de-noising techniques.

### 5.6. Wavelet De-Noising with Allan Variance

In order to combine wavelet de-noising (WD) and Allan variance under dynamic conditions, it is necessary to process the inertial sensors measures with wavelet de-noising before computing the mechanization (see Figure 3), which leads to the following question: how many levels of decomposition should be applied? In this case, the number of levels of decomposition (LOD) for the DWT are chosen based on the the spectrum of the signal after the DWT is applied. We have to consider that each level of decomposition divides the spectrum of the signal, *x*(*n*), into different sub-bands, as was explained in Section 4.5. This means that if the sampling frequency of the inertial sensor is *f_s_* = 100 Hz, after applying one LOD, we will have a spectrum between 0–25 Hz for the approximations coefficients (*A*_1_) and a spectrum between 25–50 Hz for the details coefficients (*D*_1_), considering perfect filters. Therefore, the frequency band of the wavelet de-nosing output will be limited to *f_s_*/(2×2*^k^*) for the more relevant coefficients (*A_k_*), where *k* is the level of decomposition (LOD). Since the idea is to preserve the frequency components that are associated with the motion dynamics of the land vehicle, we consider that these motion dynamics are low-frequencies components for land-vehicle applications (e.g., between 0 and 5 Hz), as is mentioned in [31]. Therefore, we evaluated the number of LOD from the one that nearly reaches 5 Hz and higher levels, i.e., considering the approximation coefficients (*A_k_*).

**Figure 17.**
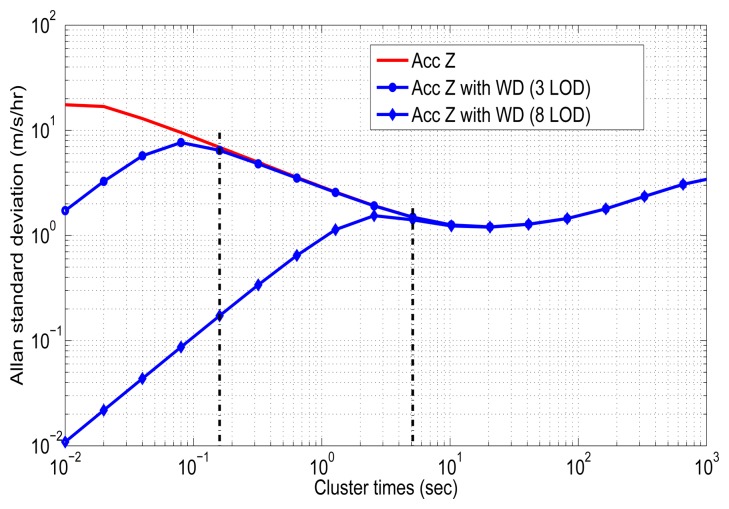
Allan variance accelerometer Z IMU 3DM-GX-25 after applying wavelet de-noising with three and eight levels of decomposition.

Thus, the test was achieved using the Matlab Wavelet Toolbox from three LOD, where the band of approximation coefficients is limited to 6.25 Hz (100/(2 × 2^3^) = 6.25), up to eight levels of decomposition, where the output band is limited to 0.1953 Hz (100/(2 × 2^8^) = 0.1953). This is taking into account that we use a sampling frequency of *f_s_* = 100 Hz for the inertial sensors. These experiments were assessed using the Daubechies family, specifically, “db4”, as the wavelet function, with soft thresholding based on Stein's Unbiased Risk Estimate (SURE), since these parameters are typically used in pre-filtering inertial sensors [4,7,31]. After selecting these wavelet de-noising parameters, the data collected in the laboratory was de-noised and, subsequently, processed with the AV algorithm. Figure 17 depicts the Allan variance standard deviation *versus* cluster times (*T*) for the *z*-axis accelerometer (red curve) after applying wavelet de-noising with three and eight levels of decomposition (blue curves). According to this plot, wavelet de-noising removed the short-term noises, while the long-term noises remain without attenuation, as was expected. It is also noticed that the higher the level of decomposition, the more high-frequency components are removed.

If we consider these two cases—the first one applying wavelet de-noising with three LOD and the second one applying eight LOD (Figure 17)—the most relevant components that correspond to the motion dynamics of the vehicle would have to be above 0.16 s and 5.12 s (vertical black dotted lines) for each case, respectively. If these components that relate to the motion dynamics are not above these cluster time values, they would be attenuated by the de-noising filters, which could degrade the INS accuracy.

**Figure 18.**
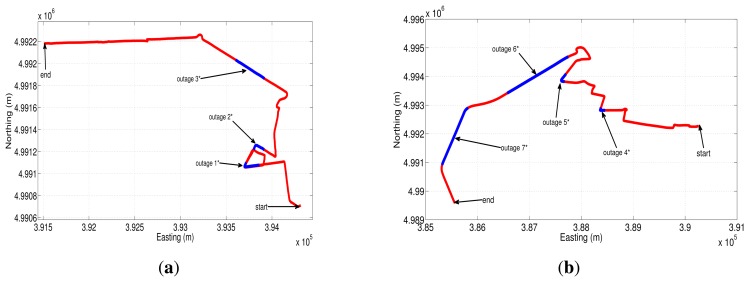
**(a)** First and **(b)** second trajectory test in Matlab with the GPS outages that were introduced intentionally to analyze the effect of wavelet de-nosing with different LOD.

Given that these motions of the vehicle are mixed with the long-term noises, a suitable LOD should be selected with the purpose of not removing relevant components that would compromise the performance of the navigation system. Therefore, to analyze the effect of wavelet de-nosing, we evaluated the enhancement accuracy of the GPS/INS solution with two vehicle tests, where a total of seven GPS outages were introduced under different dynamic conditions with a duration of 30 s and 60 s (see Figure 18), respectively. A similar procedure was achieved in [35] with a tactical-grade (medium-accuracy) and navigation-grade (high-accuracy) IMUs. The performance of the GPS/INS solution (i.e., without error models) during GPS outages with wavelet de-nosing under different LOD is summarized in Table 6. It depicts the outage number, the average speed and the maximum horizontal error for each GPS outage that was assessed. The LOD 0 corresponds to the navigation solution without applying wavelet de-noising. In the case of three LOD, we apply one level of decomposition less for *y*-axis and *z*-axis inertial sensors, since the uncorrelated noise is not so dominant for the other inertial sensors, as can be seen in the autocorrelation analysis described in Section 5.2.

Table 6 shows that the navigation solution performs slightly better for most of the GPS blockage when seven LOD are applied, compared to the navigation solution without applying wavelet de-nosing (*i.e.*, zero LOD), with an improvement of almost 4.3% in terms of horizontal positioning error.

The wavelet de-nosing parameters that provided the most significant enhance accuracy of the GPS/INS solution are summarized in Table 7. It represents the levels of decomposition where the most relevant energy associated with the motion dynamics of the vehicle remain. In this case, the most significant frequency components of the vehicle motion dynamics for the 3DM-GX3 IMU are below 0.78 Hz for *y*-axis and *z*-axis accelerometers, while for the rest of inertial sensors, it is below 0.39 Hz.

The use of Stein's Unbiased Risk Estimate (SURE) as a threshold rule helps us not to loose coefficients associated with the vehicle, since it is a conservative threshold that is usually used when small details of the signal lie in the noise range [49].

Having selected the LOD for wavelet de-noising, the long-term noises are modeled and compensated by the AV parameters obtained in Section 5.5. Overall, under dynamic conditions, wavelet de-noising will be computed for inertial sensor measurements prior to the INS mechanization (see Figure 3), and the AV model will be in charge of compensating the long-term noises. The next section explains the way the AV model and each model obtained so far is adapted into the loosely-coupled strategy.

## 6. INS Bias Model Adapted to the Loosely-Coupled KF

Having identified the random errors using AV and PSD, the parameters obtained with AV were used in the loosely-coupled GPS/INS integration scheme (Figure 3) to model the errors of accelerometers and gyros of the IMU under test. The stochastic model parameters for each sensor are taken from Table 4 and Table 5. Thus, the 3DM-GX3-25 accelerometers stochastic error *α_se_* was modeled as:

(19) $$a_{se}=WN(N)+1^{st}\;GM\;(B)+RW\;(K)$$

where the noise term associated to N is modeled as white noise (WN), the noise term associated to K as a random walk (RW), while the bias instability (B) is modeled as a first order Gauss-Markov process (first GM).

Regarding the 3DM-GX3-25 gyro stochastic error, *g_se_*, the model was defined as:

(20) $$g_{se}=WN(N)+1^{st}GM\;(B)$$

where the noise term associated to N is modeled as white noise (WN) and the bias instability (B) is modeled as a first order Gauss-Markov process (first GM). The latter noise can be modeled by a combination of Markov noise states [37], and there are also different approaches to model the bias instability noise terms; some of them are presented in [16,50]. In this case, a first order Gauss-Markov process was fitted to the flat part of the AV curve taking into account B and its correspondent correlation time (*T_c_*) (see Table 5). Regarding the noise term angle random walk (N), it presents dominant high-frequency components that have a correlation time much shorter than the sample time. Therefore, this noise is modeled as additive noise with noise variance obtained from the parameter, N (see Table 4).

On the other hand, the AR coefficients obtained from Burg's method are adapted into the KF taking parameters that were shown in Table 2. These stochastic error models were implemented in the KF according to the state-space forms that were presented in Section 3.2. Further details about IMU error state-space implementation in the Kalman filter can be found in Appendix A.1.

## 7. Results and Discussion

As explained in Section 3, we use loosely-coupled integration with feed-back, which corrects the INS error through a close-loop. The INS error dynamics equations are built in the KF, having initially nine states for position, velocity and attitude error plus additional states to estimate the bias of each sensor of the IMU.

The EKF was adapted for each designed bias model in order to evaluate the accuracy of the stochastic processes that were obtained from the previous analysis. Firstly, the two models extracted from AV\PSD were implemented, so the vector error states of the Extended Kalman Filter were augmented with six and nine states, respectively. The latter error model was combined with wavelet de-noising in order to evaluate the enhancement accuracy when Allan variance parameters and wavelet de-noising techniques are blended together. Finally, two autoregressive models were assessed augmenting EKF with six and 18 states. Table 8 summarizes the stochastic models for the 3DM-GX3 sensors and the number of states that are required in the loosely-coupled GPS/INS integration.

The EKF for the loosely-coupled integration has 15 states for two models: one is the model obtained with AV\PSD, where the bias instability (B) of both accelerometers and gyro are modeled with a first order Gauss-Markov process (GM) plus velocity\angle random walk (N), which is modeled as white noise (WN) for accelerometers and gyros, respectively. The second model with 15 states is a first order AR model. Although it is not depicted in Table 8, the AV model that was mixed with wavelet de-noising corresponds to the case of EKF with 18 states. From here on, the abbreviations 15AR, 27AR, 15AV, 18AV and 18AVWD may be used when referring to the 15 state AR, 27 state AR, 15 state AV, 18 state AV and 18 state AV with wavelet de-noising models, respectively.

**Figure 19.**
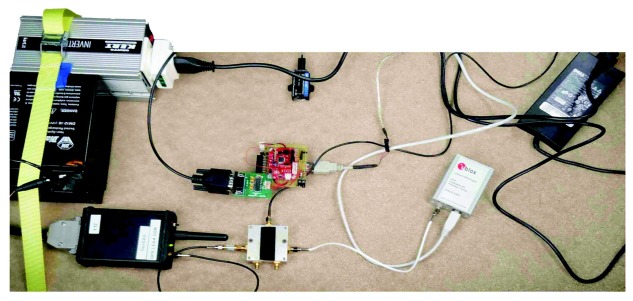
Experimental setup mounted inside the test vehicle.

In order to assess the performance of the inertial sensor error models, a car was equipped with the 3DM-GX3-25 MEMS grade IMU, which was integrated with the Sat-Surf platform with a u-blox LEA-5X receiver [46]. The specifications of the IMU can be found in Table 1. The experimental test setup that was installed inside the car is provided in Figure 19. This platform with the navigation instruments was mounted in the car rear, including the power supply that was delivered by one battery of 12 volts DC.

Two data sets were collected in urban roadways inside the city of Turin, Italy. After the data collection campaign, the loosely-coupled integration architecture with the error models presented in this paper were evaluated. Although there were no GPS outages during the campaigns, we intentionally introduced several GPS outages off line, lasting 30 s and 60 s. During an outage, the system works in prediction mode only, and the accuracy of the loosely-coupled's performance relies entirely on the INS error model and, in particular, on the INS bias model. Therefore, it is straightforward to consider different outage lengths and different vehicle's dynamic conditions in order to have a clearer answer on the accuracy of the bias models under investigation. It is really worth mentioning that since these results are based on the loosely-coupled strategy, the simulated outages have complete GPS signal blockages. The GPS/INS solution without any outages was used as a reference to compare the performance of the different error models during the simulated GPS signal blockages.

**Figure 20.**
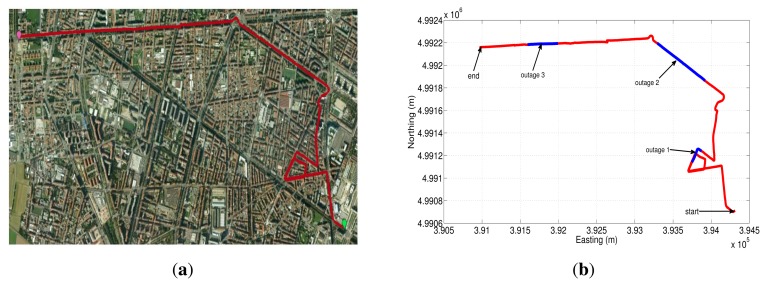
**(a)** First trajectory test in Google Earth; **(b)** first trajectory test in Matlab with the GPS outages that were introduced intentionally.

The first trajectory that was used to asses the different sensor error models is shown in Google Earth map (Figure 20(a)). This road test is part of the whole trajectory and lasts near 17.3 min; we have acquired the data from the IMU with a sampling frequency of 100 Hz. The GPS signal blockages that were intentionally introduced during post-processing are depicted in Figure 20(b) (shown as blue lines overlaid on the red trajectory), in which there are three outages with a duration of 30, 60 and 30 s for outage 1, 2 and 3, respectively. These artificial GPS outages include straight and turn portions of the trajectory in an urban roadway, which comprise typical conditions of a real GPS signal degradation inside a city.

**Figure 21.**
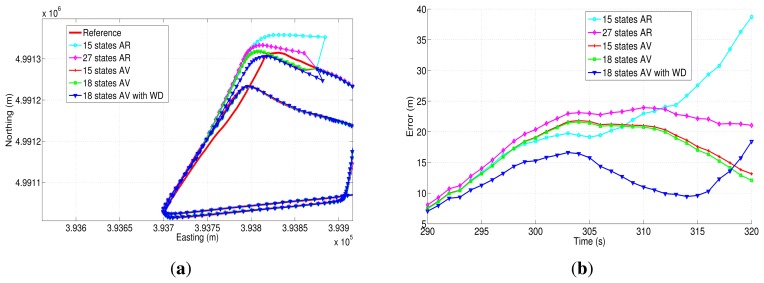
**(a)** Horizontal position during GPS outage 1; **(b)** horizontal position error during GPS outage 1.

**Figure 22.**
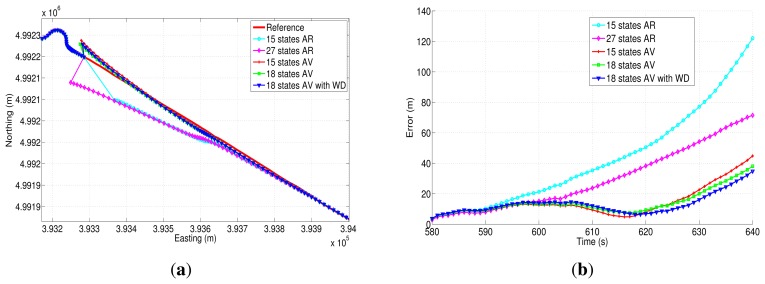
**(a)** Horizontal position during GPS outage 2; **(b)** horizontal position error during GPS outage 2.

The easting and northing position for two of the three outages (outage 1 and outage 2) are presented in Figure 21(a) and Figure 22(a), while the corresponding horizontal position error of these two outages are shown in Figure 21(b) and Figure 22(b). Table 9 summarizes the computation of the maximum and the mean horizontal position error for the error model solutions during the outages of the first trajectory. This table also shows the duration of each outage and the average speed during the three outages that were introduced in this road test.

During the first GPS outage (Figure 21(a)), there is a turn out of approximately 90 degrees; this is a challenging segment of the trajectory to evaluate the bias models, since there is an abrupt change in heading angle. From the correspondent horizontal position error (Figure 21(b)), it can be noticed that the 18AVWD model produces the minimum horizontal error, less than 15 m for almost the whole GPS outage. The mean horizontal error for the 18AVWD model is 12.56 m, while the same error parameter for the 27AR model is 19.62 m. Regarding the 15AV and 18AV state models based on Allan variance parameters, it can be seen that they are slightly similar, since the only difference is the acceleration random walk (*RW* (*K*); see Table 8), which is added in the bias model of the accelerometers. On the other hand, the 15AR model presents the worst result, having a maximum horizontal error of 38.72 m and a mean horizontal error of 20.74 m.

Figure 22(a) shows the north-easting plane for the five compared solutions during outage 2. According to Table 9, this outage lasts 60 s, and the average speed is about 42.31 km/h. This outage, introduced in a straight portion of the trajectory, shows that the 18AVWD model is better than the AR models and the other stochastic error models based on AV parameters.

**Figure 23.**
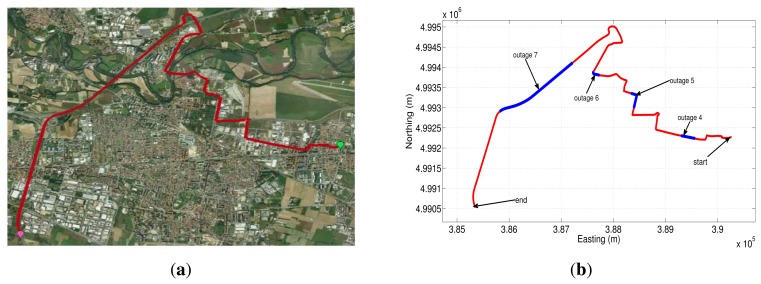
(**a**) Second trajectory test in Google Earth; (**b**) second trajectory test in Matlab with the GPS outages that were introduced intentionally.

To further validate the performance of the different stochastic error models, a second road test trajectory was collected in some urban roadways in the city of Turin; there is also a part of the path on a highway in the outskirts of the city. The road-test trajectory is 15.05 min long and is depicted in Figure 23(b).

**Figure 24.**
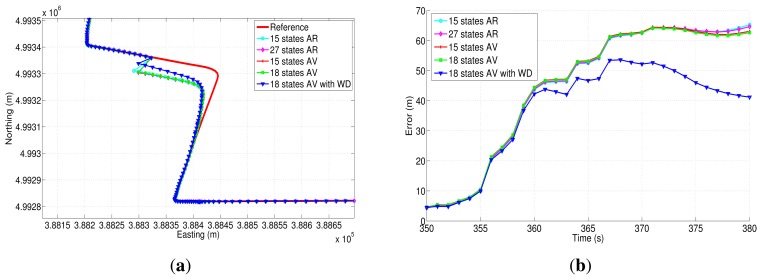
(**a**) Horizontal position during GPS outage 5; (**b**) horizontal position error during GPS outage 5.

**Figure 25.**
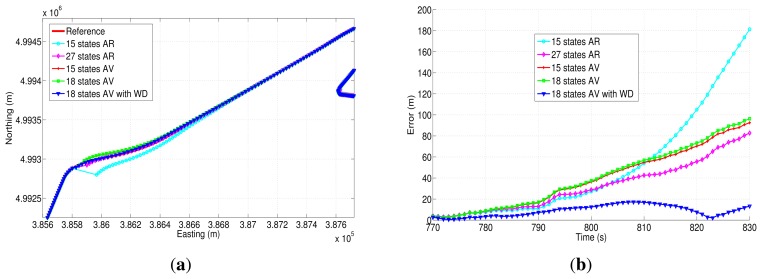
(**a**) Horizontal position during GPS outage 7; (**b**) horizontal position error during GPS outage 7.

Figure 24(a) and Figure 25(a) show two of the four GPS outages performed during the second road-test, and their respective horizontal errors can be seen in Figure 24(b) and Figure 25(b). The same as in the previous trajectory, Table 10 summarizes the mean and the maximum error for each error model analyzed, as well as the average speed and the duration of each outage that was introduced off-line.

Regarding GPS outage 5 (Figure 24(a)), it includes a turn out with an average speed of 52.25 km/h. According to the correspondent horizontal error (Figure 24(b)), it can be observed that the correction that is achieved by the 18AVWD error model is bigger with respect to the one applied through the other methods, and it has a maximum horizontal and mean position error of 53.61 m and 36.50 m, respectively, during 30 s of absence of the GPS signal. As far as GPS outage 7 is concerned, it has been simulated along a straight portion of the path, including a slight curve at the end of the outage (Figure 25(a)). This GPS blockage lasts 60 s, having an average speed of the vehicle of 112.42 km/h. This GPS outage was intended to evaluate the stochastic error models under high speed conditions, and same as in the previous GPS blockages, the 18AVWD performed better than the other models.

**Figure 26.**
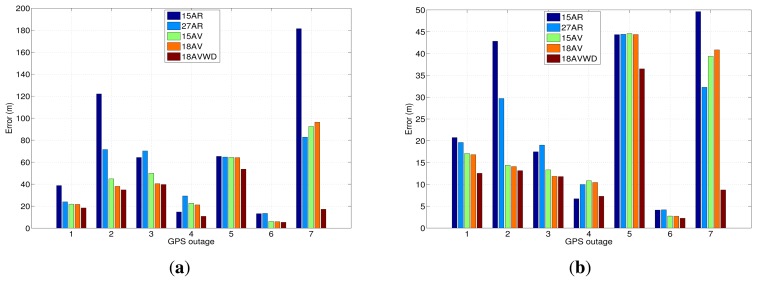
(**a**) Maximum horizontal position error for whole the GPS outage introduced in both trajectories; (**b**) mean horizontal position error for whole the GPS outage introduced in both trajectories.

In order to summarize the maximum and mean position error for both trajectories and each GPS outage performed, Figure 26 shows a comparison between the error model solutions for the seven GPS outages introduced during the test campaigns made in the city of Turin.

Overall, the model based on AV and wavelet de-noising is the one that has provided the best accuracy in most of the cases under investigation. For instance, taking into account the results that are depicted in Figure 26(a), the combination of the Allan variance parameters and wavelet de-nosing model (18AVWD) has an improvement in terms of horizontal positioning error of 50.95% over the the first order AR model (15AR) maximum horizontal position error. Furthermore, the 18AVWD provides an improvement of 48.20% over the third order AR model (27AR). Regarding the models obtained from AV, the model 18AVWD has shown an improvement of 31.89% and 26.06% over the 15AV and 18AV, respectively. Considering the mean error in horizontal positioning (Figure 26(b)), the blending of the Allan variance parameters and wavelet de-nosing (18AVWD) allowed an improvement of 39.75% over the the first order AR model (15AR), and it also has a better accuracy of almost 41.94% with respect to the third order AR model (27AR). In the same way, the 18AVWD has provided an improvement of 27.67% and 25.13% over the 15AV and 18AV, respectively.

We can also clearly appreciate how 18AV shows better results compared with 15AV in most situations where the GPS signal is not available (see Figure 26), since it offers a more adequate representation of bias-drift, according to the noise terms identified with AV and PSD (*i.e.*, the addition of the noise source associated to the acceleration random walk (K) for each of the accelerometers—18AV). Moreover, the performance of the AR models are lower than the ones obtained with AV; the explanation of this fact is commented on next.

As far as the AR technique is concerned, the main objective of using AR models and wavelet de-noising is to remove the uncorrelated noise of the inertial sensors as much as possible. In fact, if we are able to remove the main quantity of the uncorrelated noise, we can then obtain a smooth autocorrelations curve, and the noise can be modeled with an higher order Gauss-Markov process (e.g., third order AR model), with a consequent benefit on the accuracy and performance of the GPS/INS system. Unfortunately, this is not the case of the low-cost inertial sensors (MEMS IMUs) we have used in this work, since, as is shown in Section 5.2, the autocorrelation function of some of the inertial sensors after processing the data with the de-noising technique does not have a smooth autocorrelation curve, which makes the estimation of the parameters less accurate compared to the parameters obtained with AV (*i.e.*, 15AV and 18AV). Another option to get a more accurate estimation of the bias-drift can be achieved by using higher order AR models (for instance, in reference [33], the authors use an AR model with 120 states). In this case, we adopted a tradeoff between complexity and accuracy, and we selected 27 states in the AR modeling.

At last, the mixture between AV and wavelet de-noising has shown much better enhancement accuracy of the INS than the others methods presented in this work compensating for the short-term and long-term noises that affect the inertial sensors.

## 8. Conclusions and Recommendations

In this work, different stochastic error models for the measurement noise components of a MEMS-based IMU have been derived from experimental data and compared, specifically, autoregressive/wavelet de-noising models, Allan variance and Allan variance/wavelet de-noising. These stochastic models obtained from several techniques were adapted to the loosely-coupled strategy integration. Additionally, their performance was assessed in a low-cost navigation application by means of intentionally introducing several GPS outages in two trajectories collected in real urban roadways. The artificial GPS blockages were introduced in straight and curved portions of the trajectories comprising conditions of real GPS signal degradation inside a city.

Although AR processes combined with wavelet de-noising are commonly used for modeling INS stochastic errors, due to the fact that they have more modeling flexibility than first order Gauss-Markov, random walk and white noise processes, it is necessary to consider that the autocorrelation function of the IMU's raw measurements in static condition is expected to be a smooth curve (after de-noising), to use a low-order AR model, but this desired situation does not always apply for a low-cost inertial sensors (MEMs grade)

As was mentioned, the inertial sensors (MEMS grade) are affected not only by short-term noises, but also by long-term noises. Minimizing the latter is not an easy task, since these are combined with vehicle motion dynamics. In this work, we evaluated a error model that is a mixture of AV parameters and wavelet de-noising techniques (18AVWD); this model showed better performance than the other traditional methods based on AV and AR models during different GPS outages; specifically, with the 18AVWD model, we got a maximum horizontal error of 53.61 m with respect to 92.51 m (15AV), 96.32 m (18AV), 181.38 m (15AR) and 82.74 m (27AR). The 18AVWD stochastic error model uses the parameters obtained from AV to compensate for the long-term noises, while wavelet de-noising is employed to minimize the short-term noises that affect the inertial sensor of the IMU. Therefore, the wavelet de-noising technique has once again demonstrated its utility for removing the short-term noises of the inertial sensors. Nevertheless, other adaptive filtering techniques based on wavelet packet could be used in the future to get even better results, as the structure of decomposition of the sensor signal could be adapted according to the vehicle motion dynamics. It is also important to mention that, depending on the application, the selection of the decomposition level has to be carefully analyzed, due to the fact that frequency components that are associated with, e.g., vehicle motion dynamics may be eliminated after performing a de-noising technique.

It is well known that the AV technique presents drawbacks, such as: uncertainty of large clusters, so it requires large data sets to generate consistent AV curves [50]. Despite this, Allan variance uses a relatively simple procedure to characterize the random errors, and it has been successfully used in works, such as [16,40]. We plotted Allan variance together with wavelet de-nosing in the same log-log curve after applying different levels of decomposition, which has allowed us to analyze the error term attenuation and the vehicle motion dynamics. By exploiting a combined use of the AV and wavelet de-noising, we have shown how to enhance the position accuracy in a GPS/INS integrated system without excessively increasing the complexity of the INS error model.

We combined AV/Wavelet de-nosing and evaluated different levels of decomposition showing that although some vehicle motion components might be attenuated; we verified by simulation that the selected LOD provide more benefits concerning position accuracy.

Moreover, since we were dealing with a low-cost IMU, we noticed that it required many levels of decomposition to attenuate part of the uncorrelated noise and observe an enhancement in the position accuracy using wavelet de-nosing, which is not the case for high-end IMUs.

In the future, the error models analyzed in this paper could be adapted in more complex GPS/INS integration strategies, such as tightly-coupled, in order to enhance the position accuracy by using GPS estimates of pseudoranges and Doppler.

## Figures and Tables

**Table 1.** 3DM-GX3-25 IMU characteristics. Acc, accelerometer.

**IMU**	**3DM-GX3 -25**
Acc bias stability	±0.5 g for ±5 g
Acc nonlinearity	0.2%
Gyro bias stability	±0.2°/s for ±360°/s
Gyro repeatability	0.2°
Gyro nonlinearity	0.2%

**Table 2.** Autoregressive process coefficients for each inertial sensor obtained with Burg's method after wavelet de-noising with six LOD.

	*α*_**1**_	*α*_**2**_	*α*_**3**_	*β*_0_^2^(*m*/*s*^2^)^2^
Acc X	−1			2.129 * 10^−10^
	−2.582	2.166	−0.585	4.973 * 10^−13^
Acc Y	−1			1.148 * 10^−9^
	−2.564	2.136	−0.572	8.399 * 10^−12^
Acc Z	−1			1.014 * 10^−9^
	−2.564	2.136	−0.572	7.964 * 10^−12^
	*α*_**1**_	*α*_**2**_	*α*_**3**_	*β*_0_^2^(*rad*/*s*)^2^

Gyro X	−0.999			1.014 * 10^−10^
	−2.564	2.131	−0.567	2.739 * 10^−13^
Gyro Y	−0.999			7.04 * 10^−11^
	−2.565	2.133	−0.568	1.905 * 10^−13^
Gyro Z	−0.999			8.066 * 10^−11^
	−2.562	2.128	−0.566	2.181 * 10^−13^

**Table 3.** Identified error coefficients for accelerometers and gyro of the 3DM-GX3 IMU with PSD.

	**Velocity Random Walk (N)** $\left(m/s/(\sqrt{h}\right)$	**Bias Instability (B)** (*m*/*s*/*h*)	**Acceleration Random Walk (K)** (*m*/*s*/*h*^3/2^)
Acc X	0.045	4.6447	168.60
Acc Y	0.044	4.6700	26.66
Acc Z	0.047	1.7733	14.60
1–4	**Angle Random Walk (N)** $\left(\text{deg}/\sqrt{h}\right)$	**Bias Instability (B)** (*deg*/*h*)	**Rate Random Walk (K)** (*deg*/*h*^3/2^)

Gyro X	2.297	43.438	
Gyro Y	1.937	39.614	
Gyro Z	2.058	30.705	

**Table 4.** Identified error coefficients for accelerometers and gyro of the 3DM-GX3 IMU with AV.

	**Velocity Random Walk (N)** $(m/s/(\sqrt{h}\text{)}$	**Bias Instability (B)** (*m*/*s*/*h*)	**Acceleration Random Walk (K)** (*m*/*s*/*h*^3/2^)
Acc X	0.045 ± 0.00023	5.1581 ± 0.0370	166.30 ± 4.6398
Acc Y	0.045 ± 0.00022	4.5507 ± 0.0506	24.95 ± 2.8368
Acc Z	0.047 ± 0.00050	1.8336 ± 0.0524	13.53 ± 1.8685
	**Angle Random Walk (N)** $\left(\text{deg}/\sqrt{h}\right)$	**Bias Instability (B)** (*deg*/*h*)	**Rate Random Walk (K)** (*deg*/*h*^3/2^)

Gyro X	2.420 ± 0.0974	44.533 ± 5.14	
Gyro Y	1.988 ± 0.0565	38.810 ± 2.51	
Gyro Z	2.164 ± 0.0599	31.717 ± 2.29	

**Table 5.** Identified correlation time, (*Tc*), for the bias instability (B) and standard deviation for each inertial sensor of the 3DM-GX3 IMU.

	**Acc X**	**Acc Y**	**Acc Z**	**Gyro X**	**Gyro Y**	**Gyro Z**
Correlation time *T_c_* (*s*)	4.56	7.26	20.74	490.89	623.25	735.17
Standard deviation (*m*/*s*^2^ − *rad*/*s*)	0.0068	0.0065	0.0063	0.0055	0.0045	0.0048

**Table 6.** Maximum horizontal position error during GPS outages before and after applying wavelet de-noising.

**Outage**	**Duration (seg)**	**Average Speed (km/h)**	**Levels of Decomposition**		

			**0**	**3**	**7**

			**max (m)**	**max (m)**	**max (m)**
1*	30	25.16	85.12	85.13	76.08
2*	30	18.86	162.98	162.67	157.73
3*	30	42.53	189.67	189.69	188.82
4*	30	23.56	52.20	52.20	51.62
5*	30	39.32	54.03	54.01	43.99
6*	60	103.36	232.54	232.74	217.28
7*	60	122.73	279.22	279.03	274.74

**Table 7.** Wavelet de-noising parameters for each sensor under kinematic conditions.

	**LOD**	**Frequency Limit for***A_k_***Coefficients (Hz)**	**Thresholding**
Acc X	7	0.39	soft, SURE
Acc Y	6	0.78	soft, SURE
Acc Z	6	0.78	soft, SURE
Gyro X	7	0.39	soft, SURE
Gyro Y	7	0.39	soft, SURE
Gyro Z	7	0.39	soft, SURE

**Table 8.** Number of states in the loosely coupled integration architecture for different error models.

	**15 States**	**18 States**	**27 States**
AV\PSD *α_se_*	*WN*(*N*)	*WN*(*N*)	
	+	+	
	*GM*(*B*)	*GM*(*B*)	
		+	
		*RW*(*K*)	

AV\PSD *g_se_*	*WN*(*N*)	*WN*(*N*)	
	+	+	
	*GM*(*B*)	*GM*(*B*)	

AR *α_se_*\*g_se_*	1^st^ *order AR*		3^rd^*order AR*

**Table 9.** Maximum and mean horizontal position error during GPS outages for trajectory 1. 15AR, 15 state AR; 27AR, 27 state AR; 15AV, 15 state AV; 18AV, 18 state AV; 18AVWD, 18 state AV with wavelet de-noising

**Outage**	**Duration (sec)**	**Average Speed (km/h)**	**Stochastic error model**									

			**15AV**		**18AV**		**18AVWD**		**15AR**		**27AR**	

			**mean (m)**	**max (m)**	**mean (m)**	**max (m)**	**mean (m)**	**max (m)**	**mean (m)**	**max (m)**	**mean (m)**	**max (m)**
1	30	23.58	17.11	21.84	16.82	21.60	12.56	18.42	20.74	38.72	19.62	23.94
2	60	42.31	14.41	44.84	14.10	38.04	13.16	34.81	42.82	122.07	29.70	71.49
3	30	47.60	13.34	49.97	11.86	40.41	11.80	39.69	17.48	64.26	19.02	70.21

**Table 10.** Maximum and mean horizontal position error during GPS outages for trajectory 2.

**Outage**	**Duration (sec)**	**Average speed (km/h)**	**Stochastic error model**									
			**15AV**		**18AV**		**18AVWD**		**15AR**		**27AR**	
			**mean (m)**	**max (m)**	**mean (m)**	**max (m)**	**mean (m)**	**max (m)**	**mean (m)**	**max (m)**	**mean (m)**	**max (m)**
4	30	33.60	10.85	22.70	10.44	21.13	7.29	10.81	6.72	14.72	9.99	29.26
5	30	52.25	44.57	64.46	44.36	64.17	36.50	53.61	44.34	65.28	44.43	64.65
6	30	20.44	2.74	6.19	2.71	5.91	2.25	5.32	4.12	13.17	4.18	13.41
7	60	112.42	39.40	92.51	40.85	96.32	8.74	17.20	49.62	181.38	32.27	82.74

## A. Appendix

### A.1. IMU Error State-Space Implementation in the KF

The equations that represent the error dynamics in the n-frame for the loosely-coupled approach are given by position error, (*δ**ṟ*^*n*^), velocity error, (*δ**v̱^n^*), and attitude error, (*δψ̱*^*n*^). The description of the transition matrix for these nine states is detailed in [10,23], and the derivation of these error equations can be found in [14,51]. In order to evaluate the models, it is necessary to increase these nine states, (*δṟ^n^*, *δv̱^n^*, *δψ̱^n^*), with the IMU error states. For illustrative purposes, in this section, we only present the state-space form of the model associated with 18 states calculated with Allan variance and the 27 state model, where the third order AR model is adopted.

### IMU error state-space for the 18 state AV model

To include the bias of the inertial sensors (*i.e.*, Equations (19) and (20)), the transition matrix in the discrete time is augmented from the initial nine states, as in Equation (21):

(21) $$A_{k,9x9}=\left[\begin{array}{ccc} I_{3\times 3}-\text{diag}({\underline{\beta }}_{a}\Delta t) & 0_{3\times 3} & 0_{3\times 3}\\ 0_{3\times 3} & I_{3\times 3}-\text{diag}({\underline{\beta }}_{g}\Delta t) & 0_{3\times 3}\\ 0_{3\times 3} & 0_{3\times 3} & I_{3\times 3} \end{array}\right]$$

where Δ*t* is the sampling time of the INS *i.e.*, 0.01 *s*, while *β̱_a_* and *β̱_g_* correspond to the reciprocal of correlation time, (*T_c_*), presented in Table 5 for accelerometers and gyros, respectively. This transition matrix was described for one inertial sensor in Equation (7); in the case of the three accelerometers, the expression, *diag*(*β̱_a_* δ*t*), is given by:

(22) $$\text{diag}({\underline{\beta }}_{a}\Delta t)=\left[\begin{array}{ccc} \beta_{ax} & 0 & 0\\ 0 & \beta_{ay} & 0\\ 0 & 0 & \beta_{az} \end{array}\right]\cdot \Delta t$$

The complete error states after adapting Equation (21) into the first nine state of the KF is presented in Equation (23):

(23) $$\delta \underline{x}={\left[\begin{array}{llllll} \delta {\underline{r}}^{n} & \delta {\underline{v}}^{n} & \delta {\underline{\Psi }}^{n} & \delta {\underline{b}}_{a,bi} & \delta {\underline{b}}_{g,bi} & \delta {\underline{b}}_{a,k} \end{array}\right]}^{T}$$

where *δḇ_a_*_,_*_bi_* and *δḇ_g_*_,_*_bi_* are the bias-drift associated to the first order GM process for accelerometers and gyros, respectively, while *δḇ_a_*_,_*_k_* is the bias-drift of the accelerometers that represents the random walk process.

The design matrix, *G*, for the 18 error states AV can be written as:

(24) $$G=\left[\begin{array}{lllll} 0_{3\times 3} & 0_{3\times 3} & 0_{3\times 3} & 0_{3\times 3} & 0_{3\times 3}\\ C_{b}^{n} & 0_{3\times 3} & 0_{3\times 3} & 0_{3\times 3} & 0_{3\times 3}\\ 0_{3\times 3} & -C_{b}^{n} & 0_{3\times 3} & 0_{3\times 3} & 0_{3\times 3}\\ 0_{3\times 3} & 0_{3\times 3} & I_{3\times 3} & 0_{3\times 3} & 0_{3\times 3}\\ 0_{3\times 3} & 0_{3\times 3} & 0_{3\times 3} & I_{3\times 3} & 0_{3\times 3}\\ 0_{3\times 3} & 0_{3\times 3} & 0_{3\times 3} & 0_{3\times 3} & I_{3\times 3} \end{array}\right]$$

where $C_{b}^{n}$ is the frame rotation matrix from the body to the n-frame [14,27].

(25) $$C_{b}^{n}=\left[\begin{array}{ccc} c\theta c\psi & -c\phi s\psi +s\phi s\theta c\psi & s\phi s\psi +c\phi s\theta c\psi \\ c\theta s\psi & c\phi c\psi +s\phi s\theta s\psi & -s\phi c\psi +c\phi s\theta s\psi \\ -s\theta & s\phi c\theta & c\phi c\theta \end{array}\right]$$

where “sin” and “cos” are shortly denoted as “s” and “c”, respectively. The variables, *φ*, *θ* and *ψ*, correspond to the Euler angles (Roll, Pitch and Yaw).

The noise covariance matrix Q of the model is:

(26) $$Q=\left[\begin{array}{ccccc} \text{diag}({\underline{q}}_{a,n}) & 0_{3\times 3} & 0_{3\times 3} & 0_{3\times 3} & 0_{3\times 3}\\ 0_{3\times 3} & \text{diag}({\underline{q}}_{g,n}) & 0_{3\times 3} & 0_{3\times 3} & 0_{3\times 3}\\ 0_{3\times 3} & 0_{3\times 3} & \text{diag}({\underline{q}}_{a,bi}) & 0_{3\times 3} & 0_{3\times 3}\\ 0_{3\times 3} & 0_{3\times 3} & 0_{3\times 3} & \text{diag}({\underline{q}}_{b,bi}) & 0_{3\times 3}\\ 0_{3\times 3} & 0_{3\times 3} & 0_{3\times 3} & 0_{3\times 3} & \text{diag}({\underline{q}}_{a,k}) \end{array}\right]$$

where *q̱_a,n_*, *q̱_a,bi_* and *q̱_a_*_,_*_k_* are the spectral densities of the process driving noises for each noise term that are used to model the bias-drift of the accelerometers (*i.e.*, WN, RW and first order GM process). Similarly, *q̱_g_*_,_*_n_* and *q̱_g_*_,_*_bi_* are the noise variance quantities that will be used within the KF to model the bias-drift of the gyros (*i.e.*, WN and first order GM process). All these quantities are computed based on the parameters that were obtained with the AV technique (see Table 4 and Table 5). In order to describe the relationship between the parameters obtained from the experiments (*i.e.*, N, B and K), and the noise process that are modeled (*i.e.*, WN, first order GM process and RW), we take as an example the *x*-axis accelerometer, so the spectral density in discrete time of the process driving noises of a WN process can be expressed as:

(27) $$q_{ax,n}=\sigma_{W\;N_{ax}}^{2}/\Delta t=N_{ax}^{2}/(3600*\Delta t)$$

where $\sigma_{W\;N_{ax}}^{2}$ is the variance of the white noise process and N is the velocity random walk associated to the *x*-axis accelerometer from Table 4. The spectral density, *q_ax_*_,_*_b__i_*_,_ in discrete time for the first order GM process is given by [29]:

(28) $$q_{ax,bi}=\sigma_{GM_{ax}}^{2}(1-e^{-2\Delta t/T_{c,ax}})$$

where *T_c_*_,_*_ax_* is the correlation time from Table 5 and $\sigma_{GM_{ax}}^{2}$ is the covariance of the first GM process that can be determined by means of the bias instability parameter for the *x*-axis accelerometer (*B_ax_*) from Table 4.

(29) $$\sigma_{GM_{ax}}=B_{ax}*0.664/3600$$

The spectral density, *q_ax_*_,_*_bi_*, in discrete time for the random walk process can be expressed as:

(30) $$q_{ax,k}=\sigma_{RW_{ax}}^{2}*\Delta t=K_{ax}^{2}*\Delta t/{\left(3600\right)}^{3}$$

where $\sigma_{RW_{ax}}^{2}$ is the noise covariance of the RW process and *K_ax_* is the acceleration random walk for the *x*-axis accelerometer from Table 4.

### IMU error state-space for the 27 states with third order AR models

The transition matrix in the discrete time used to augment the KF with a bias-drift modeled as a third order AR process for each inertial sensors can be described by Equation (31):

(31) $$A_{k,18x18}=\left[\begin{array}{cccccc} 0_{3\times 3} & I_{3\times 3} & 0_{3\times 3} & 0_{3\times 3} & 0_{3\times 3} & 0_{3\times 3}\\ 0_{3\times 3} & 0_{3\times 3} & I_{3\times 3} & 0_{3\times 3} & 0_{3\times 3} & 0_{3\times 3}\\ -\text{diag}({\underline{\alpha_{3}}}^{a}) & -\text{diag}({\underline{\alpha }}_{2}^{a}) & -\text{diag}({\underline{\alpha }}_{1}^{a}) & 0_{3\times 3} & 0_{3\times 3} & 0_{3\times 3}\\ 0_{3\times 3} & 0_{3\times 3} & 0_{3\times 3} & 0_{3\times 3} & I_{3\times 3} & 0_{3\times 3}\\ 0_{3\times 3} & 0_{3\times 3} & 0_{3\times 3} & 0_{3\times 3} & 0_{3\times 3} & I_{3\times 3}\\ 0_{3\times 3} & 0_{3\times 3} & 0_{3\times 3} & -\text{diag}({\underline{\alpha }}_{3}^{g}) & -\text{diag}({\underline{\alpha }}_{2}^{g}) & -\text{diag}({\underline{\alpha }}_{1}^{g}) \end{array}\right]$$

where ${\underline{\alpha }}_{1}^{a}$, ${\underline{\alpha }}_{2}^{a}$ and ${\underline{\alpha }}_{3}^{a}$ are vectors with the coefficients for the three accelerometers, while ${\underline{\alpha }}_{1}^{a}$, ${\underline{\alpha }}_{2}^{a}$ and ${\underline{\alpha }}_{3}^{a}$ are the vectors with the coefficients for the three gyros. This transition matrix was described for one inertial sensor in Equation (10); in the case of the three accelerometers, the expression, $\text{diag}({\underline{\alpha }}_{1}^{a})$, is given by:

(32) $$\text{diag}({\underline{\alpha }}_{1}^{a})=\left[\begin{array}{ccc} \alpha_{1}^{ax} & 0 & 0\\ 0 & \alpha_{1}^{ay} & 0\\ 0 & 0 & \alpha_{1}^{az} \end{array}\right]$$

The complete error states of the KF will have 27 states, which are given by:

(33) $$\delta \underline{x}={\left[\begin{array}{lllllllll} \delta {\underline{r}}^{n} & \delta {\underline{v}}^{n} & \delta {\underline{\psi }}^{n} & \delta {\underline{b}}_{a,b1} & \delta {\underline{b}}_{a,b2} & \delta {\underline{b}}_{a,b3} & \delta {\underline{b}}_{g,b1} & \delta {\underline{b}}_{g,b2} & \delta {\underline{b}}_{g,b3} \end{array}\right]}^{T}$$

where *δ̱b_a_*_,_*_b_*_1_, *δḇ_a,b_*_2_ and *δḇ_a,b_*_3_ are the nine states associated to the third order AR models of the three accelerometer, while *δḇ_g,b_*_1_, *δḇ_g,b_*_2_ and *δḇ_g,b_*_3_ are the nine states associated to the third order AR models of the three gyros.

The design matrix, *G*, for the 27 error states based on the third AR models can be written as:

(34) $$G=\left[\begin{array}{llll} 0_{3\times 3} & 0_{3\times 3} & 0_{3\times 3} & 0_{3\times 3}\\ C_{b}^{n} & 0_{3\times 3} & 0_{3\times 3} & 0_{3\times 3}\\ 0_{3\times 3} & -C_{b}^{n} & 0_{3\times 3} & 0_{3\times 3}\\ 0_{3\times 3} & 0_{3\times 3} & 0_{3\times 3} & 0_{3\times 3}\\ 0_{3\times 3} & 0_{3\times 3} & 0_{3\times 3} & 0_{3\times 3}\\ 0_{3\times 3} & 0_{3\times 3} & I_{3\times 3} & 0_{3\times 3}\\ 0_{3\times 3} & 0_{3\times 3} & 0_{3\times 3} & 0_{3\times 3}\\ 0_{3\times 3} & 0_{3\times 3} & 0_{3\times 3} & 0_{3\times 3}\\ 0_{3\times 3} & 0_{3\times 3} & 0_{3\times 3} & I_{3\times 3} \end{array}\right]$$

In this case, the noise covariance matrix, *Q*, of the model is:

(35) $$Q=\left[\begin{array}{llll} \text{diag}({\underline{q}}_{a,n}) & 0_{3\times 3} & 0_{3\times 3} & 0_{3\times 3}\\ 0_{3\times 3} & \text{diag}({\underline{q}}_{g,n}) & 0_{3\times 3} & 0_{3\times 3}\\ 0_{3\times 3} & 0_{3\times 3} & \text{diag}({\underline{q}}_{a,b}) & 0_{3\times 3}\\ 0_{3\times 3} & 0_{3\times 3} & 0_{3\times 3} & \text{diag}({\underline{q}}_{g,b}) \end{array}\right]$$

where *q̱_a,n_*, *q̱_g,n_* are the same spectral densities of the white noise processes described in the 18 states AV models, while *q̱_a,b_* and *q̱_g,b_* are the the spectral densities of the third order AR processes for each inertial sensor. In the case of the *y*-axis accelerometer, the spectral density in discrete time is by given by:

(36) $$q_{ay,b}=\beta_{0}^{2}$$

where *β*_0_ is the standard deviation of the AR process.

## References

[1] Weston J., Titterton D. (2000). Modern inertial navigation technology and its application. Electron. Commun. Eng. J..

[2] Shin E.H. (2005). Estimation Techniques for Low-Cost Inertial Navigation. Ph.D. Thesis.

[3] El-Sheimy N., Nassar S., Noureldin A. (2004). Wavelet de-noising for IMU alignment. IEEE Aerosp. Electron. Syst. Mag..

[4] Skaloud J., Bruton A.M., Schwarz K.P. (1999). Detection and filtering of short-term (1/*f*) noise in inertial sensors. J. Navig..

[5] El-Sheimy N., Nassar S., Schwarz K.P., Noureldin A. (2004). Modeling inertial sensor errors using autoregressive (AR) models. Navigation.

[6] Ramalingam R., Anitha G., Shanmugam J. (2009). Microelectromechanical systems inertial measurement unit error modelling and error analysis for low-cost strapdown inertial navigation system. Def. Sci. J..

[7] Noureldin A., Armstrong J., El-Shafie A., Karamat T., McGaughey D., Korenberg M., Hussain A. (2012). Accuracy enhancement of inertial sensors utilizing high resolution spectral analysis. Sensors.

[8] El-Sheimy N., Hou H., Niu X. (2008). Analysis and modeling of inertial sensors using allan variance. IEEE Trans. Instrum. Meas..

[9] Vaccaro R., Zaki A. (2012). Statistical modeling of rate gyros. IEEE Trans. Instrum. Meas..

[10] Aggarwal P., Syed Z., Noureldin A., El-Sheimy N. (2010). MEMS-Based Integrated Navigation.

[11] (1998). IEEE Standard Specification Format Guide and Test Procedure for Single-Axis Interferometric Fiber Optic Gyros.

[12] Titterton D., Weston J. (2004). Strapdown Inertial Navigation Technology.

[13] Farrell J.A., Barth M. (1999). The Global Positioning System & Inertial Navigation.

[14] Schultz C.E. (2006). INS and GPS Integration. M.Sc. Thesis.

[15] Skog I., Händel P. Calibration of a MEMS Inertial Measurement Unit.

[16] Moafipoor S., Bock L., Fayman J., Mader G., de Jonge P. Development and Assessment of a Low Dynamic Vehicle Navigation System.

[17] Hou H. (2004). Modeling Inertial Sensors Errors Using Allan Variance. M.Sc. Thesis.

[18] El-Diasty M., Pagiatakis S. (2009). A rigorous temperature-dependent stochastic modelling and testing for MEMS-based inertial sensor errors. Sensors.

[19] El-Diasty M., Pagiatakis S. (2008). Calibration and stochastic modelling of inertial navigation sensor errors. J. Glob. Position. Syst..

[20] Flenniken W., Wall J., Bevly D. Characterization of Various IMU Error Sources and the Effect on Navigation Performance.

[21] Wall J.H., Bevly D.M. Characterization of Inertial Sensor Measurements for Navigation Perfor-mance Analysis.

[22] Babu R., Wang J. Real-Time Data Analysis of Ultra-Tight GPS/INS Integration.

[23] Falco G., Einicke G.A., Malos J.T., Dovis F. (2012). Performance analysis of constrained loosely coupled GPS/INS integration solutions. Sensors.

[24] Li Y., Wang J., Rizos C., Mumford P., Ding W. Low-Cost Tightly Coupled GPS/INS Integration Based on a Nonlinear Kalman Filtering Design.

[25] Godha S. (2006). Performance Evaluation of Low-Cost MEMS-Based IMU Integrated with GPS for Land Vehicle Navigation Application. M.Sc. Thesis.

[26] Gelb A. (1974). Applied Optimal Estimation.

[27] Grewal M.S., Weill L.R., Andrews A.P. (2007). Global Positioning Systems, Inertial Navigation and Integration.

[28] Rogers R. (2000). Applied Mathematics in Integrated Navigation Systems.

[29] Brown R.G., Hwang P.Y.C. (1997). Introduction to Random Signals and Applied Kalman Filtering with MATLAB Exercises.

[30] Mohamed A.H. (1999). Optimizing the Estimation Procedure in INS/GPS Integration for Kinematic Applications. Ph.D. Thesis.

[31] Noureldin A., Karamat T., Eberts M., El-Shafie A. (2009). Performance enhancement of MEMS-based INS/GPS integration for low-cost navigation applications. IEEE Trans. Veh. Technol..

[32] Waegli A., Skaloud J. Assessment of GPS/MEMS-IMU Integration Performance in Ski Racing.

[33] Georgy J., Noureldin A., Korenberg M., Bayoumi M. (2010). Modeling the stochastic drift of a MEMS-based gyroscope in gyro/odometer/GPS integrated navigation. IEEE Trans. Intell. Transp. Syst..

[34] Park M., Gao Y. (2008). Error and performance analysis of MEMS-based inertial sensors with a low-cost GPS receiver. Sensors.

[35] Nassar S. (2003). Improving the Inertial Navigation System (INS) Error Model for INS and INS/DGPS Applications. Ph.D. Thesis.

[36] Wong R.V.C., Schwarz K.P., Cannon M.E. (1998). High-accuracy kinematic positioning by GPS-INS. Navigation.

[37] (1999). IEEE Standard Specification Format Guide and Test Procedure for Linear, Single-Axis, Non-Gyroscopic Accelerometers.

[38] El-Diasty M., Pagiatakis S. (2010). A frequency-domain INS/GPS dynamic response method for bridging GPS outages. J. Navig..

[39] Naranjo C.C. (2008). Analysis and Modeling of MEMS Based Inertial Sensors. M.Sc. Thesis.

[40] Hidalgo J., Poulakis P., Khler J., Del-Cerro J., Barrientos A. (2012). Improving planetary rover attitude estimation via MEMS sensor characterization. Sensors.

[41] Zhang X., Li Y., Mumford P., Rizos C. Allan Variance Analysis of Error Characteristics of MEMS Inertial Sensors for an FPGA-Based GPS/INS System.

[42] Kang C.W., Kang C.H., Park C.G. Wavelet De-Noising Technique for Improvement of the Low Cost MEMS-GPS Integrated System.

[43] Wu X., Duan L., Chen W. A Kalman Filter Approach Based on Random Drift Data of Fiber Optic Gyro.

[44] (2010). 3DM-GX3-25 Technical Product Overview Data Sheet.

[45] (2010). 3DM-GX3-25 Data Communications Protocol.

[46] Navsas Navigation Signal Analysis and Simulation, Sat-Surfer Training board & Software Suite for GNSS training.

[47] Heinzel G., Rudiger A., Schilling R. (2002). Spectrum and Spectral Density Estimation by the Discrete Fourier Transform(DFT), Including a Comprehensive List of Window Functions and Some New At-Top Windows.

[48] Niu X., Nasser S., Goodall C., El-Sheimy N. (2007). A universal approach for processing any MEMS inertial sensor configuration for land-vehicle navigation. J. Navig..

[49] Michel M., Yves M.G.O., Poggi J.M. (1996). Wavelet Toolbox: for Use with MATLAB—User's Guide.

[50] Yuksel Y., El-Sheimy N., Noureldin A. Error Modeling and Characterization of Environmental Effects for Low Cost Inertial MEMS Units.

[51] Solimeno A. (2007). Low-Cost INS/GPS Data Fusion with Extended Kalman Filter for Airborne Applications. M.Sc. Thesis.

